# Integrative analysis of rumen microbiota and host multi-organ interactions underlying feed conversion efficiency in Hu sheep

**DOI:** 10.1186/s40104-025-01333-3

**Published:** 2026-02-03

**Authors:** Xiaowei Jia, Yuanxin Zhang, Boya Tian, Guijie Zhang, Shengyong Mao, Wenxi Qian, Daming Sun, Junhua Liu

**Affiliations:** 1 Ruminant Nutrition and Feed Engineering Technology Research Center, College of Animal Science and Technology, Nanjing Agricultural University, Nanjing, 210095 China; 2 Laboratory of Gastrointestinal Microbiology, National Center for International Research On Animal Gut Nutrition, Nanjing Agricultural University, Nanjing, 210095 China; 3 College of Animal Science and Technology, Tarim University, Alar, 843300 China; 4 College of Animal Science, Ningxia University, Yinchuan, 750021 China

**Keywords:** Feed efficiency, Host transcriptomics, Rumen microbiota, Sheep

## Abstract

**Background:**

Rumen microbiota drive fermentation and contribute to variation in feed efficiency among ruminants, yet the underlying host–microbe mechanisms remain poorly understood. This study explores how rumen microbes shape feed conversion efficiency (FCR) through integrated interactions with multiple host organs.

**Results:**

We applied a multi-omics strategy—combining rumen metagenomics and host multi-organ transcriptomics—in Hu sheep with divergent FCR. From a uniform cohort of 150 weaned male Hu lambs, 13 low-FCR (LFCR) and 13 high-FCR (HFCR) individuals were selected for integrated analyses. LFCR sheep exhibited greater growth performance and higher ruminal propionate concentrations compared with HFCR animals. The ruminal microbiomes were enriched in *Saccharofermentans* and Succinivibrionaceae_UBA2804, and showed functional convergence on amino acid biosynthesis, central carbon metabolism, and propionate-oriented fermentation in LFCR sheep. Carbohydrate-active enzyme profiles indicated that LFCR animals favored fiber- and starch-associated modules (GH126, CBM27, EPS-GT), whereas HFCR animals were enriched in host-glycan and uronic acid–degrading families (CE14, GH89, PL15). Hydrogen metabolism highlighted a clear dichotomy: LFCR animals redirected H₂ toward propionate and sulfate reduction, while HFCR animals retained greater butyrate-producing and methanogenic capacity. Transcriptomic profiling across rumen epithelium, liver, and muscle identified tissue-specific regulatory modules. Only the liver showed strong enrichment of carbohydrate metabolism, with a complete glycogen turnover and glucose export system (*GYS2*, *PYGL*, *PGM2*, *G6PC1*) and pathways linking microbial short-chain fatty acids to gluconeogenesis. In contrast, muscle efficiency modules were dominated by contractile and cytoskeletal genes (e.g., *MYL2*, *TNNC1*, *TPM3*), reflecting optimized energy expenditure rather than substrate metabolism. No efficiency-associated modules were detected in the rumen epithelium, consistent with its role in propionate absorption rather than metabolism.

**Conclusions:**

The rumen microbiota of LFCR sheep possess highly efficient capacities for volatile fatty acid and amino acid synthesis, thereby enhancing energy utilization at its source. The resulting propionate further promotes hepatic gluconeogenesis, directly supplying energy for muscle cell growth and ultimately improving FCR. Thus, co-metabolism between rumen microbiota and the liver provides energy for muscle cell growth and is a key determinant of improved feed efficiency.

**Supplementary Information:**

The online version contains supplementary material available at 10.1186/s40104-025-01333-3.

## Background

The growing global population and rising demand for animal-derived protein are placing unprecedented pressure on livestock production systems to improve efficiency and sustainability [1, 2]. Feed remains the most significant cost in ruminant production, often exceeding 65% of total inputs. As a result, improving feed efficiency (FE) has become a key objective in animal science, with the dual goals of increasing profitability and reducing environmental impact [3, 4]. In China, sheep farming has experienced rapid intensification to meet the growing demand for mutton. Under standardized feeding regimes—where animals are provided with identical diets and management practices—considerable individual variation in FE still exists [5–7]. This variation offers an opportunity to uncover biological mechanisms that underpin nutrient utilization efficiency, thereby enabling the identification of biomarkers for genetic selection or the development of targeted feed additives [8, 9].

The rumen microbiota plays a central role in the breakdown of complex plant materials and in the release of nutrients that support ruminant growth [10, 11]. Through microbial fermentation, structural polysaccharides and proteins are converted into volatile fatty acids (VFAs), amino acids, vitamins, and gases such as hydrogen and methane [12–14]. VFAs—particularly acetate, propionate, and butyrate—provide up to 80% of the ruminant host’s metabolizable energy [15]. Therefore, the composition and function of the rumen microbial community are key determinants of energy harvesting and overall feed efficiency [16, 17]. Recent metagenomic studies have shown that microbial functional pathways, especially those related to carbohydrate and amino acid metabolism, are strongly associated with FE traits such as feed conversion ratio (FCR) and residual feed intake (RFI), even when overall community diversity remains unchanged [6, 18, 19]. These findings suggest that functional capacity, rather than richness or diversity alone, is central to efficiency differences.

Current research on FE has primarily examined interactions between the rumen microbiota and the ruminal epithelium [3, 20, 21]. However, the rumen functions mainly as an absorptive organ for VFAs, whereas microbial crude protein produced in the rumen is largely absorbed in the small intestine. Furthermore, acetate and propionate absorbed across the rumen wall are predominantly metabolized in the liver and peripheral tissues [22]. Thus, while rumen microbiota–epithelium interactions are important, studies addressing the metabolic integration of rumen-derived substrates with extra-ruminal organs, particularly the liver and muscle, remain limited. Dissecting this rumen-liver-muscle metabolic axis is therefore critical for elucidating how microbial metabolites are integrated into host energy metabolism and growth regulation.

Therefore, the present study aims to investigate the mechanisms by which rumen microbiota influence feed conversion efficiency in ruminants, with a particular focus on the co-metabolism between the rumen and liver and its downstream impact on muscle growth. By integrating microbial composition, metabolic potential, and host gene expression, we aimed to (i) identify microbial and host signatures linked to FE, (ii) elucidate nutrient utilization and energy partitioning mechanisms, and (iii) provide insights into rumen microbiota and host multi-organ interactions in ruminants. We seek to provide a systems-level understanding of microbe–host interactions underlying FE, thereby offering new targets for nutritional interventions and genetic improvement in ruminant production.

## Methods

### Experimental animals and design

A total of 150 healthy, approximately 3-month-old, weaned male Hu lambs with complete pedigree records were used in this study. All animals were raised under standardized feeding and housing conditions at a commercial meat sheep farm in Huzhou, Zhejiang Province, China. Lambs were individually housed in indoor pens with slatted floors (200 cm × 60 cm) and fed diets formulated according to the national feeding standards for meat sheep and goats [23]. The detailed ingredients and nutrient composition of the basal diet have been described previously [24]. Animals were fed twice daily (08:00 and 17:00) with free access to water, and feed refusal was maintained at 10%–15%. Dry matter intake (DMI) was recorded daily, and body weights (BW) were measured biweekly to calculate average daily gain (ADG), average daily feed intake (ADFI) and FCR.

**Fig. 1:**
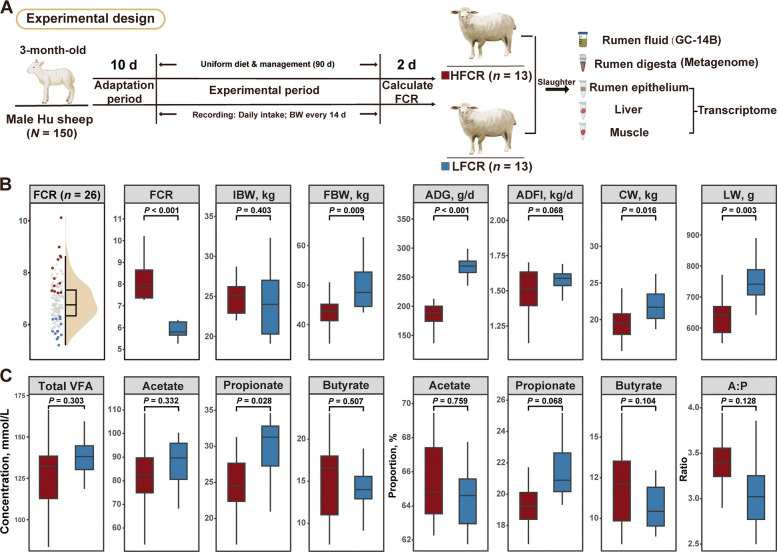
Experimental design and phenotypic traits of Hu sheep with divergent feed efficiency. **A** Experimental timeline and sample collection for metagenomic (rumen digesta) and transcriptomic (rumen epithelium, liver, muscle) analyses in male Hu sheep (*n* = 26; HFCR, high feed conversion ratio, *n* = 13; LFCR, low feed conversion ratio, *n* = 13). **B** Growth performance differences between groups. FCR, feed conversion ratio; IBW, initial body weight; FBW, final body weight; ADG, average daily gain; ADFI, average daily feed intake; CW, carcass weight; LW, liver weight. **C** Comparison of rumen fermentation parameters. VFA, volatile fatty acids; A:P, acetate-to-propionate ratio. Boxes depict the interquartile range with medians; whiskers extend to 1.5 × IQR

The feeding trials were conducted in two cohorts under identical management and dietary conditions. Within each cohort, animals with implausible values attributable to recording errors or abnormal growth were excluded. From the remaining population, lambs with the highest and lowest FCR values were selected to represent the extreme phenotypes, yielding a total of 26 animals (13 LFCR and 13 HFCR; Fig. 1A). The experimental protocol was approved by the Animal Care and Use Committee of Nanjing Agricultural University (Approval No. NJAU.20220903N09).

### Sample collection

On the final day of the feeding trial, selected lambs were weighed 3 h after feeding to obtain final body weight (FBW) and then humanely slaughtered by electrical stunning followed by exsanguination, in accordance with institutional animal welfare guidelines. Carcass (CW) and liver weights (LW) were recorded, and samples of rumen digesta, rumen epithelium, liver, and longissimus dorsi muscle were collected immediately after slaughter. The entire rumen digesta was transferred into a sterile container and thoroughly mixed to ensure homogeneity before sampling. The homogenized digesta was aliquoted into 5 mL cryovials, snap-frozen in liquid nitrogen, and stored at − 80 °C until DNA extraction. An additional portion was filtered through four layers of sterile gauze to obtain rumen fluid, which was stored at − 20 °C for VFA analysis using gas chromatography (GC-14B, Shimadzu, Japan) [25]. Rumen epithelium was sampled from the ventral sac. A piece of rumen wall (approximately 5 cm × 5 cm) was excised, gently rinsed three times with ice-cold sterile phosphate-buffered saline (PBS) to remove residual digesta. The epithelial layer was then carefully separated from the underlying muscle layer, cut into small pieces, and placed into cryovials. All tissue samples, including rumen epithelium, liver, and longissimus dorsi muscle, were immediately snap-frozen in liquid nitrogen and stored at − 80 °C until RNA extraction.

### DNA extraction and metagenomic sequencing

After thorough vortexing, 200 mg of rumen digesta was transferred into a centrifuge tube containing 100 mg of 0.1 mm zirconia beads and 1 mL of InhibitEX buffer. Mechanical disruption was performed using a Mini-Beadbeater-1 (Biospec, USA) at 4,800 r/min for 20 s per cycle, repeated four times, following the bead-beating procedure of Yu and Morrison [26]. Microbial genomic DNA was then purified using the E.Z.N.A.^®^ Stool DNA Kit (Omega Bio-Tek, Norcross, GA, USA) according to the manufacturer’s instructions. DNA integrity was evaluated by 1% agarose gel electrophoresis, and concentration and purity were determined using a NanoDrop ND-1000 spectrophotometer (Thermo Fisher Scientific, Wilmington, USA). Samples with an A_260_/A_280_ ratio of 1.95–2.10 were considered acceptable and stored at − 80 °C until sequencing. Metagenomic libraries were prepared using the TruSeq DNA PCR-Free Library Preparation Kit (Illumina, USA) with an average insert size of 350 bp, and paired-end sequencing (2 × 150 bp) was performed on the Illumina NovaSeq 6000 platform.

### Metagenomic assembly and gene catalog construction

Raw reads were quality-trimmed using Trimmomatic (v.0.33) to remove adapter sequences and low-quality bases, and read quality was evaluated using FastQC [27]. Clean reads were mapped to the sheep reference genome (*Ovis aries*, Oar_v3.1), major dietary component genomes (*Zea mays* GCA_902167145.1, *Arachis hypogaea* GCA_003086295.2, *Glycine max* GCA_000004515.4), and the human genome (*Homo sapiens*, GCA_000001405.28) using BWA-MEM (v.0.7.12) to remove host- and diet-derived contaminants [28]. Unmapped reads were retained for downstream analysis.

Decontaminated reads from each sample were independently assembled de novo using MEGAHIT (v.1.1.1) with a minimum contig length of 500 bp [29]. Open reading frames (ORFs) were predicted from each sample’s assembled contigs with Prodigal (v.2.6.3) in metagenomic mode, only ORFs located on contigs ≥ 1,500 bp were retained [30]. The resulting ORF sets from all samples were merged and clustered using CD-HIT-EST v.4.8.1 (parameters: -c 0.95, -aS 0.9, -G 0, -n 9), and genes sharing ≥ 95% nucleotide identity and ≥ 90% alignment coverage were retained as representative sequences in the non-redundant gene catalog [31, 32].

### Taxonomic classification and functional annotation

The non-redundant gene catalog was translated into protein sequences using EMBOSS transeq [33]. The resulting protein catalog was annotated using DIAMOND v0.9.22 BLASTP (e-value ≤ 1 × 10^−^^5^, top 10 hits) against the GTDB-corrected NCBI-NR (GTDB, release 220) and KEGG (release 202304) databases [34, 35]. Carbohydrate-active enzymes (CAZymes) were annotated separately using HMMER v3.2.1 based on profile hidden Markov models (HMMs) from the CAZy database (v13) [36, 37]. Hydrogenases were identified by aligning protein sequences against HydDB (e-value < 1 × 10^–50^; coverage ≥ 90%; identity ≥ 50%) and categorized into [NiFe]-, [FeFe]-, and [Fe]-hydrogenase groups [38]. The best hits were retained for downstream analyses.

High-quality reads from each sample were mapped to the non-redundant gene catalog using BWA-MEM, and gene abundances were calculated using the transcripts per million (TPM) method [39]. Taxonomic profiles were derived from gene-level annotations by aggregating the TPM values of all annotated genes assigned to each lineage from the phylum to genus level. For visualization, taxa detected at ≥ 1% relative abundance at the phylum level or ≥ 0.1% at the genus level in at least one sample were displayed individually, with the remainder grouped as “Others”. Functional profiles were constructed by aggregating TPM values to KEGG Orthologs (KOs), CAZy families (GH, GT, PL, CE, CBM, AA), and hydrogenases ([NiFe], [FeFe], [Fe]). Annotations from KOs, CAZy, and hydrogenases were integrated with taxonomic information to construct function-by-taxon abundance matrices.

For each differentially abundant functional feature, TPM were summed across all LFCR or HFCR samples within each genus to obtain group-level abundances. The genus-level difference was then calculated as:

$$\Delta_g={sum}_{LFCR,g}-{sum}_{HFCR,g}$$

The total change for the feature was defined as:

$${\Delta }_{total}=\sum_{g}|{\Delta }_{g }|$$

Finally, the proportional contribution of each genus was expressed as:

$${Contribution}_g\left(\%\right)=\frac{\left|\Delta_g\right|}{\Delta_{total}}\times100$$

The sign of ${\Delta }_{g}$ was used to distinguish the direction of enrichment (LFCR vs. HFCR). Genera contributing < 20% were pooled as “Others”, and only the top five per direction (LFCR > HFCR vs. HFCR > LFCR) were retained for visualization.

### RNA extraction, library construction, and sequencing

Approximately 100 mg of frozen tissue (rumen epithelium, liver, and muscle) from a subset of seven Hu sheep (HFCR = 7; LFCR = 7) randomly selected from the 26 animals used for metagenomic analysis was powdered with a sterilized mortar and pestle under liquid nitrogen. Total RNA was extracted using TRIzol Reagent (Life Technologies) following the manufacturer’s protocol. RNA quantity and purity were measured with a NanoDrop 2000, and integrity was assessed using an Agilent 2100 Bioanalyzer. High-quality RNA (RNA integrity number > 7.0) was used for library preparation. mRNA was enriched by oligo(dT) selection (Hieff NGS™ mRNA Isolation Master Kit, Yeasen), followed by cDNA synthesis, end repair, 3′ adenylation, adaptor ligation, and PCR indexing according to the manufacturer’s protocol. The transcriptome libraries were sequenced on the Illumina NovaSeq 6000 platform (paired-end 150 bp).

### Read processing, alignment, and quantification for tissue transcriptome analysis

Raw reads were adapter- and quality-trimmed and screened for ambiguous bases. Read quality metrics (Q20/Q30, GC content, duplication) were assessed with FastQC v0.11.9. Clean reads were aligned to the sheep reference genome (*Ovis aries*, Oar_v3.1) using HISAT2 v2.0.4 with recommended settings [40]. Transcripts were assembled and quantified with StringTie v2.2.1 in reference-guided (RABT) mode [41]. Gene expression was summarized as fragments per kilobase of transcript per million mapped reads (FPKM) [39]. Unless otherwise specified, genes with FPKM > 1 in a given tissue were retained for downstream analysis.

### Functional enrichment and WGCNA

For each tissue, expressed genes (FPKM > 1) were subjected to functional enrichment using clusterProfiler v4.12.0, including KEGG analyses [42]. To relate transcriptomes to phenotypic traits, WGCNA was run independently per tissue [43]. The soft-thresholding power (β) was chosen using pickSoftThreshold to achieve a scale-free topology fit index (*R*^2^) ≥ 0.85. Networks were constructed from the adjacency matrix, transformed to a topological overlap matrix (TOM), and modules were detected by the dynamic tree cut algorithm with subsequent merging when appropriate. Module eigengenes were correlated with tissue-specific phenotypic traits to identify functionally relevant modules. Spearman correlations were calculated between representative microbial functional genes and eigengenes of host WGCNA modules. In addition, after identifying five hepatic genes involved in carbohydrate and energy metabolism, we performed RT-qPCR analysis to validate their expression patterns.

### Statistical analysis and data visualization

Comparisons between HFCR and LFCR groups for continuous phenotypes (initial body weight (IBW), FBW, CW, LW, FCR, ADG, ADFI, and microbial fermentation parameters) were performed using two-sided Student’s *t*-tests after testing for normality (Shapiro–Wilk) and homogeneity of variance (Levene’s test). Welch’s *t*-test was applied when variances were unequal.

Microbial community structure was evaluated at the species level by principal coordinates analysis (PCoA) based on Bray–Curtis dissimilarities. Group separation was assessed using PERMANOVA (*vegan::adonis2*, 999 permutations), with homogeneity of multivariate dispersion tested by *vegan::betadisper*. ANOSIM (999 permutations) was applied as a complementary test [44]. Alpha diversity indices (Shannon and Simpson) were calculated from species-level abundance matrices using vegan v2.5.6, and between-group differences in diversity indices and taxonomic abundances were evaluated using Wilcoxon rank-sum tests. Group differences in the relative abundance of microbial functional features (e.g., KEGG Orthologs, CAZy families, hydrogenase classes) were tested with Wilcoxon rank-sum tests.

Figures (box plots, bar plots, scatter plots, heatmaps) were generated in R (version as in the computing environment) using ggplot2, pheatmap, gridExtra, ggsci, ggpubr, and egg [45–50]. Statistical significance was set at *P* < 0.05, and statistical trends were reported when 0.05 ≤ *P* < 0.10, unless otherwise stated.

## Results

### Animal growth performance and rumen fermentation parameters

At the end of the trial, the LFCR group showed a markedly lower FCR than the HFCR group (*P* < 0.001), while IBW did not differ (*P* = 0.403). In contrast, FBW (*P* = 0.009), ADG (*P* < 0.001), CW (*P* = 0.016), and LW (*P* = 0.003) were all greater in LFCR. The ADFI tended to be higher in LFCR (*P* = 0.068, Fig. 1B).

For rumen fermentation parameters, total VFA, acetate, and butyrate concentrations showed no differences between groups (*P* > 0.05, Fig. 1C), whereas propionate concentration was higher in LFCR (*P* = 0.028, Fig. 1C). For VFA molar proportions, propionate tended to be higher in LFCR (*P* = 0.068), with acetate, butyrate, and the acetate: propionate (A:P) ratio showing no differences (*P* > 0.05, Fig. 1C).

### Rumen metagenome overview

Given the between-group differences in rumen fermentation, we performed shotgun metagenomic sequencing of rumen digesta to characterize microbial composition and functional potential. Across 26 samples, 4,040,968,582 raw reads were obtained (155,421,869 ± 14,915,958 per sample; Additional file 1: Table S1). After adapter trimming and quality control, 3,943,780,776 clean reads remained (151,683,876 ± 14,571,120; Additional file 1: Table S2). Subsequent removal of host- and feed-derived reads retained 1,260,595,262 read pairs (48,484,433 ± 7,256,228; Additional file 1: Table S3). De novo assembly with MEGAHIT yielded 20,807,473 contigs (800,287 ± 173,012; Additional file 1: Table S4). Clustering predicted genes at 95% identity generated a non-redundant microbial gene catalog comprising 6,371,471 genes.

Domain-level taxonomic profiling showed that the metagenomes were dominated by Bacteria (92.37%), followed by Eukaryota (5.57%), Viruses (1.09%), and Archaea (0.56%) (Additional file 1: Table S5). Functional annotation assigned 2,972,176 (48.8%) to KOs and 416,454 (6.8%) to CAZy families.

### Microbial community structure and composition

**Fig. 2:**
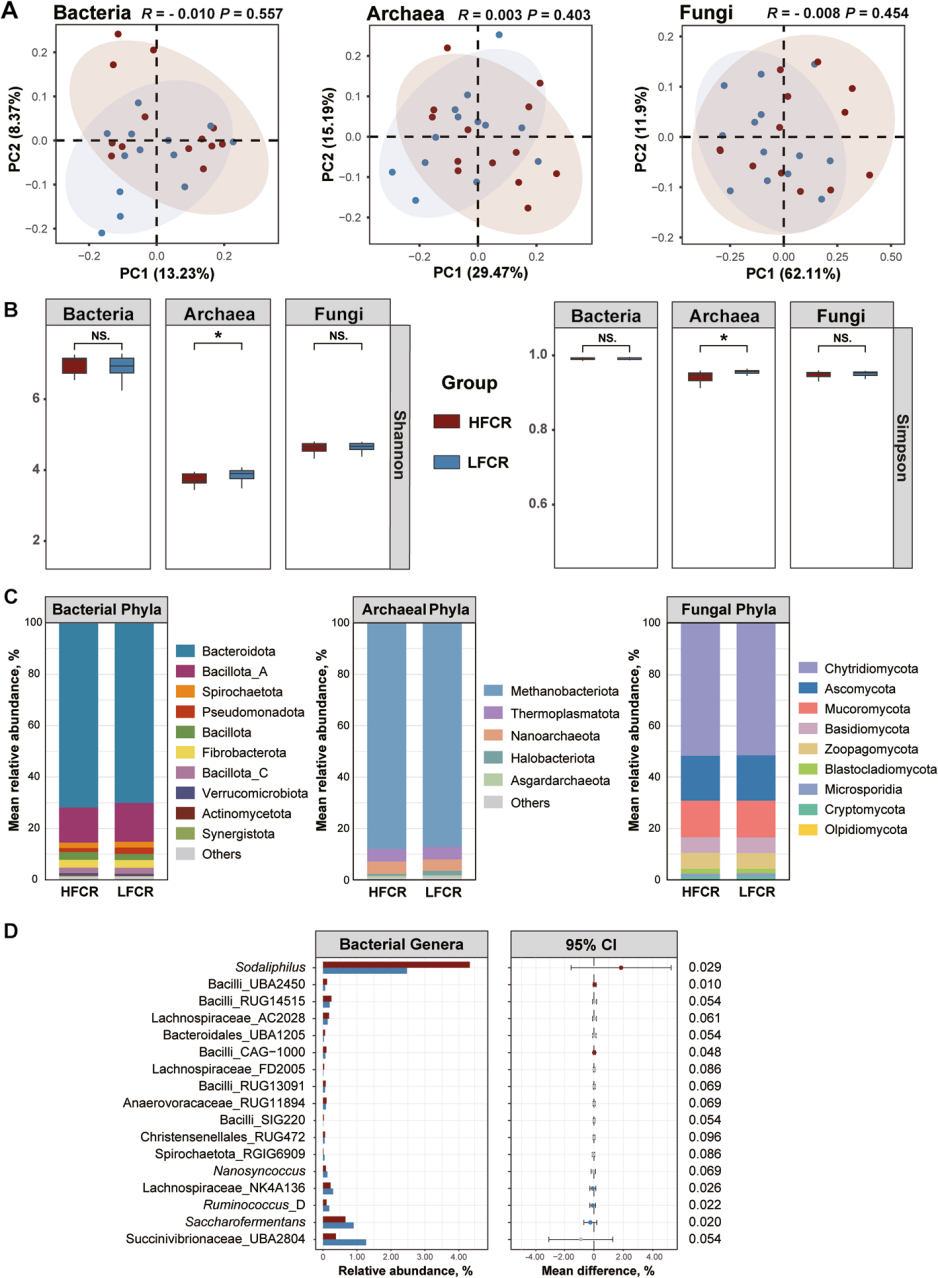
Microbial community diversity and composition in Hu sheep with divergent feed efficiency. **A** Principal coordinates analysis (PCoA) based on Bray–Curtis dissimilarities of bacterial, archaeal, and fungal communities at the species level. **B** Alpha diversity (Shannon and Simpson indices calculated at the species level) for bacteria, archaea, and fungi *P < 0.05; NS., not significant. **C** Phylum composition within domains; phyla ≥ 1% in any sample shown, others pooled as “Others”; abundances renormalized per domain. **D** Genus-level summaries for bacteria: left, group-wise mean relative abundance; right, LFCR–HFCR difference with 95% CI (confidence interval). Filled points: *P* < 0.05; open: 0.05 ≤ *P* < 0.10

Beta diversity (PCoA based on Bray–Curtis) tested by ANOSIM showed no significant separation between HFCR and LFCR for bacteria, archaea, or fungi (*P* > 0.05; Fig. 2A). In alpha diversity, bacteria and fungi showed no between-group differences (Shannon, Simpson; *P* > 0.05), whereas archaea differed between groups (Shannon, Simpson; *P* < 0.05).

At the phylum level, community compositions were comparable with no significant differences (*P* > 0.05; Fig. 2C). At the genus level, several taxa differed between groups (*P* < 0.05; Fig. 2D): in bacteria, *Saccharofermentans*, *Ruminococcus*_D, and Lachnospiraceae_NK4A136 were more abundant in LFCR, whereas Bacilli_UBA2450, Bacilli_CAG-1000, and *Sodaliphilus* were enriched in HFCR (Fig. 2D).

### Functional characteristics of rumen microbiota with different feed efficiency

A total of 3,835,390 microbial genes were annotated, of which 2,972,176 were assigned to 3,256 KOs and 416,454 to 375 CAZyme families.

**Fig. 3:**
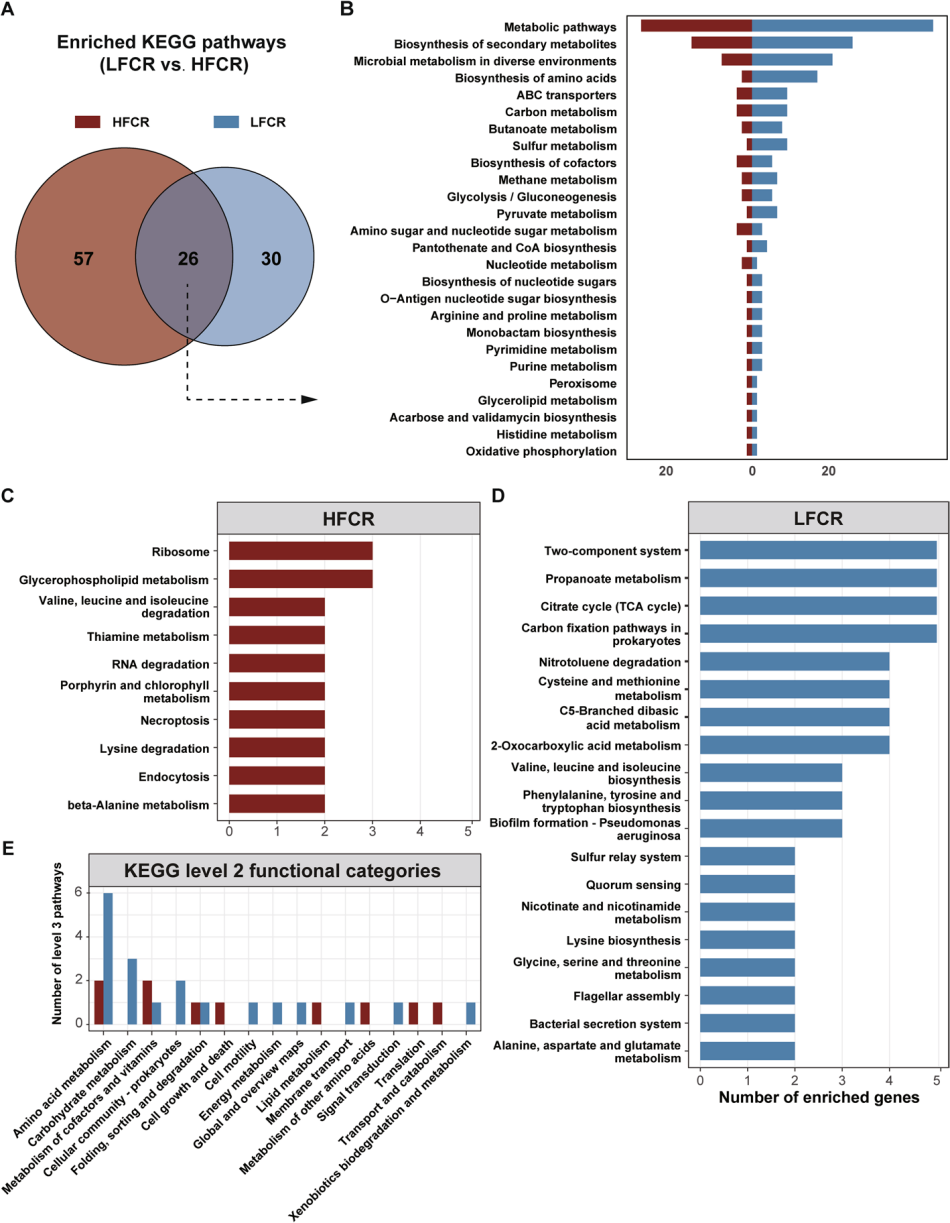
KEGG pathway differences between LFCR and HFCR. **A** Venn diagram of pathways enriched in each group based on differentially abundant KOs (*P* < 0.05); numbers indicate shared and group-specific counts. **B** Shared pathways ranked by the number of enriched KOs. **C **and** D** Group-specific pathways (HFCR-only in C; LFCR-only in D) restricted to those supported by ≥ 2 enriched KOs; bars show KO counts per pathway. **E** Group-specific signals summarized to KEGG level-2 categories. KEGG, Kyoto Encyclopedia of Genes and Genomes; KO, KEGG Orthology

At the KEGG level, 105 KOs differed between groups (58 higher in LFCR; 47 higher in HFCR; *P* < 0.05). Mapping these to pathways identified 56 LFCR-enriched and 83 HFCR-enriched pathways; 26 were shared, while 30 and 57 were unique to LFCR and HFCR, respectively (Fig. 3A).

Among the shared pathways, LFCR was predominantly enriched in amino acid biosynthesis, carbon metabolism, butanoate metabolism, and sulfur metabolism (Fig. 3B). For group-specific pathways (≥ 2 enriched KOs), LFCR was enriched in the tricarboxylic acid (TCA) cycle, propanoate metabolism, and carbon fixation in prokaryotes, whereas HFCR was dominated by ribosome and glycerophospholipid metabolism (Fig. 3C and D). Collapsing these to KEGG level 2 categories revealed a stronger representation of amino acid and carbohydrate metabolism in LFCR (Fig. 3E), indicating coordinated carbon–nitrogen utilization. At KEGG level 1, no enrichment was detected in the Organismal Systems category for LFCR (Additional file 2: Fig. S1A and B). Given that amino acid biosynthesis accounted for 25% (14/58) of all LFCR-enriched KOs—the largest single functional category—we examined this process in detail.

### Differential enrichment of amino-acid metabolic pathways

**Fig. 4:**
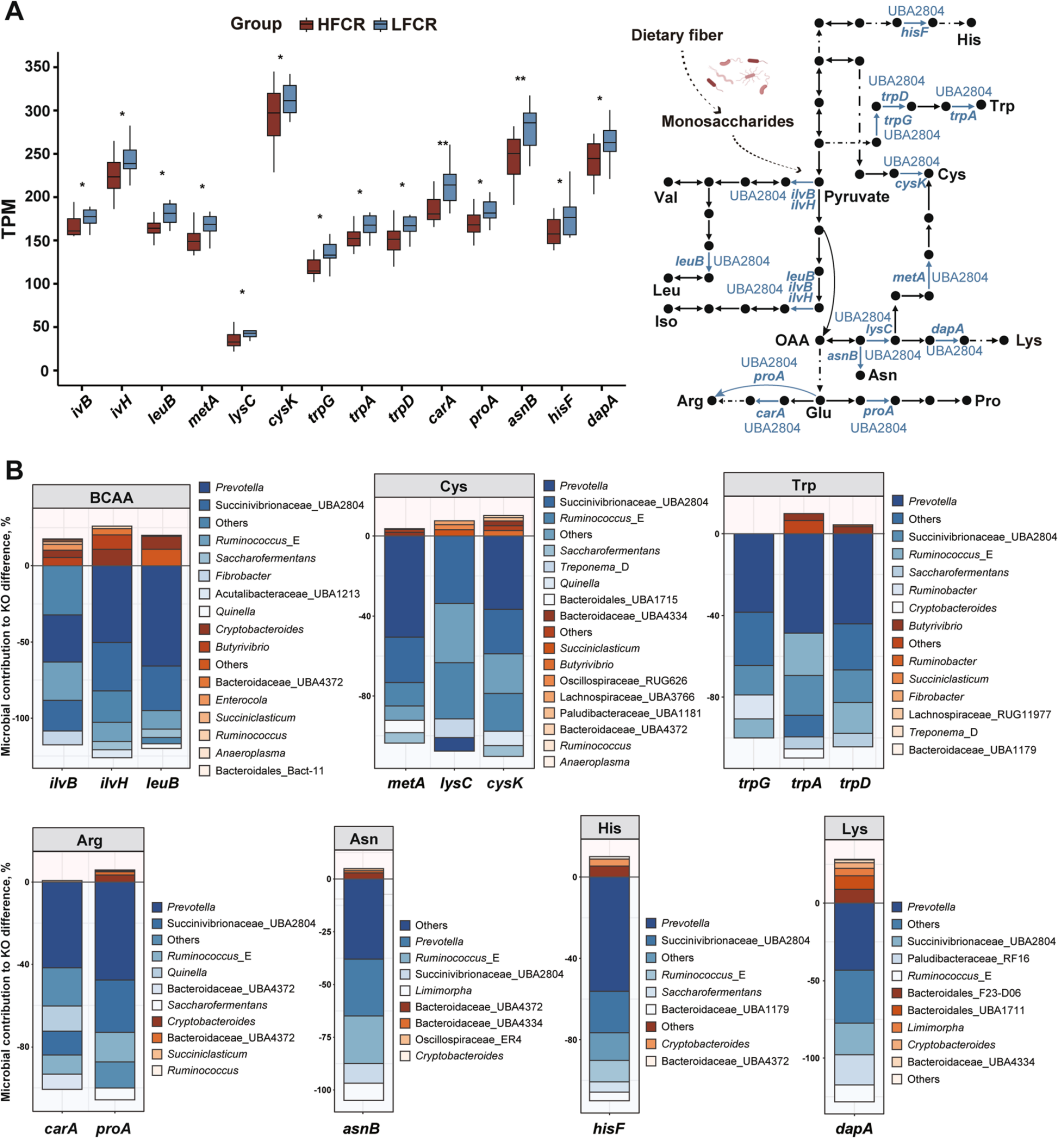
Amino-acid metabolism and taxonomic attribution in the rumen microbiome. **A** Left: Boxplots of TPM for the 14 KEGG orthologs (KOs) within amino-acid metabolism that differed between groups. Significance by Wilcoxon rank-sum: *P* < 0.05 (*), *P* < 0.01 (**). Right: Schematic showing the positions of these KOs within central amino-acid metabolic pathways, with major contributing genera annotated, including Succinivibrionaceae_UBA2804 (abbreviated as UBA2804 in the figure). His, histidine; Trp, tryptophan; Cys, cysteine; Val, valine; Leu, leucine; Ile, isoleucine; OAA, oxaloacetic acid; Lys, lysine; Asn, asparagine; Arg, arginine; Glu, glutamate; Pro, proline. **B** Microbial contribution to KO differences for each pathway module. For each KOs, only the top five genera per direction are shown, with the remainder merged into “Others” (including both < 20% contributors and additional genera beyond the top five). Labels are displayed for contributions ≥ 3%. TPM, transcripts per million; BCAA, valine, leucine and isoleucine biosynthesis; Cys, cysteine, methionine and glutathione metabolism; Trp, tryptophan biosynthesis; Arg, arginine and proline metabolism; Asn, Alanine, aspartate and glutamate metabolism; His, histidine biosynthesis; Lys, lysine metabolism

Fourteen KOs related to amino acid metabolism were significantly enriched in LFCR (*P* < 0.05), spanning pathways including arginine/proline metabolism, cysteine/methionine metabolism, glutathione metabolism, branched-chain amino acid biosynthesis (valine, leucine, isoleucine), lysine metabolism, tryptophan biosynthesis, and histidine biosynthesis (Fig. 4A).

Taxonomic assignment linked these KOs primarily to *Prevotella*, Succinivibrionaceae_UBA2804, *Ruminococcus*_E, *Treponema*_D, *Saccharofermentans*, and *Cryptobacteroides* (Fig. 4B). Consistent with genus-level profiles (Fig. 2D), *Saccharofermentans* and Lachnospiraceae_NK4A136 were more abundant in LFCR (*P* < 0.05), while Succinivibrionaceae_UBA2804 tended to be higher (0.05 ≤ *P* < 0.10).

### Differential carbohydrate degradation in rumen microbiota

To further resolve functional differences in carbohydrate metabolism, we profiled CAZymes. From assembled contigs, 396,952 non-redundant CAZyme genes were identified, spanning 140 glycoside hydrolase (GH) families, 83 glycosyltransferase (GT) families, 33 polysaccharide lyase (PL) families, 17 carbohydrate esterase (CE) families, 90 carbohydrate-binding module (CBM) families, and 12 auxiliary activity (AA) families (Additional File 1: Table S6). Class-level distributions did not differ between groups (Additional file 2: Fig. S2).

**Fig. 5:**
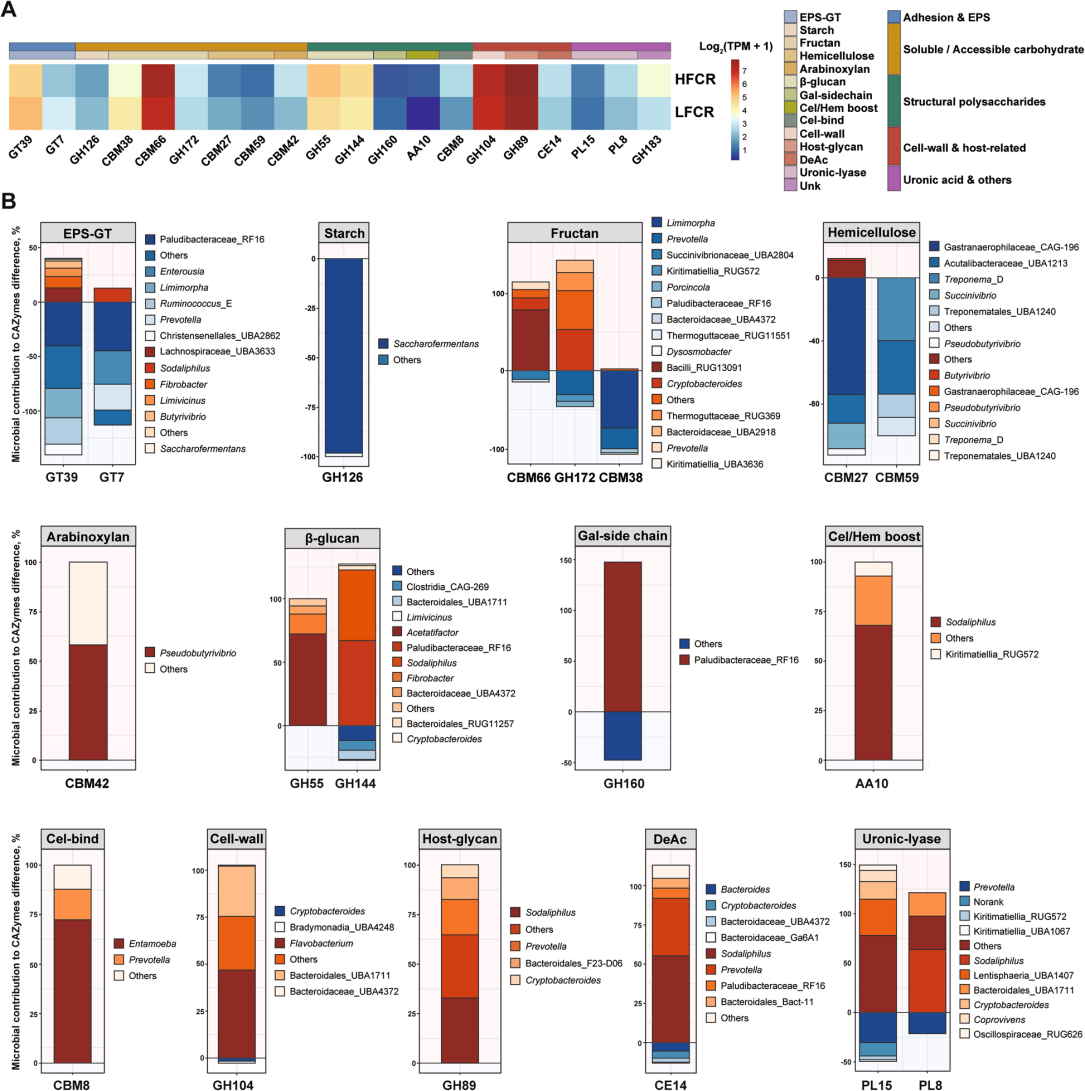
Family-level differences in CAZyme profiles and their taxonomic attribution between HFCR and LFCR groups. **A** Relative abundance of CAZyme families compared between HFCR and LFCR. **B** Genus-level attribution of differential CAZyme families. Stacked bar plots indicate the proportional contribution of microbial genera to the observed KO differences, plotted using the same approach as in Fig. 4. GT, glycosyltransferase family; GH, glycoside hydrolase family; CBM, carbohydrate-binding module family; PL, polysaccharide lyase family; AA, auxiliary activity family; CE, carbohydrate esterase family; EPS-GT, exopolysaccharide glycosyltransferase; Gal-side chain, galactose side-chain; Cel/Hem boost, cellulose/hemicellulose boost; Cell-bind, cell-binding module; Cell-wall, cell-wall–related module; Host-glycan, host-derived glycan module; DeAc, deacetylase; Uronic-lyase, uronic-acid lyase; Unk, unknown

CAZymes were consolidated into 14 functional categories grouped into five major categories (Fig. 5A). At the family level, LFCR animals exhibited higher abundances of exopolysaccharide glycosyltransferases (GT39, GT7; *P* < 0.05), starch-active GH126 (*P* < 0.05) and hemicellulose-associated CBM27 (*P* < 0.05), contributed mainly by Paludibacteraceae_RF16, *Saccharofermentans*, *Limimorpha*, Gastranaerophilaceae_CAG-196, and *Treponema*_D (Fig. 5B). HFCR animals showed greater abundance of fructan-related CBM66 and GH172 (*P* < 0.05), arabinoxylan-binding CBM42 (*P* < 0.05), β-glucan hydrolases GH55 and GH144 (*P* < 0.05), cellulose-binding CBM8 (*P* < 0.05), oxidative AA10 (*P* < 0.05), deacetylase CE14 (*P* < 0.05), host-glycan hydrolase GH89 (*P* < 0.05), and uronic-acid metabolism enzymes PL15 and PL8 (*P* < 0.05). These were predominantly derived from Bacilli_RUG13091, *Pseudobutyrivibrio*, *Acetatifactor*, Paludibacteraceae_RF16, *Sodaliphilus*, *Entamoeba*, and *Flavobacterium* (Fig. 5B).

### Fermentation routes and hydrogen sink in the rumen microbiome

Beyond polysaccharide breakdown, we profiled KOs and hydrogenase genes associated with VFA synthesis and hydrogen turnover to capture functional differences in fermentation pathways.

**Fig. 6:**
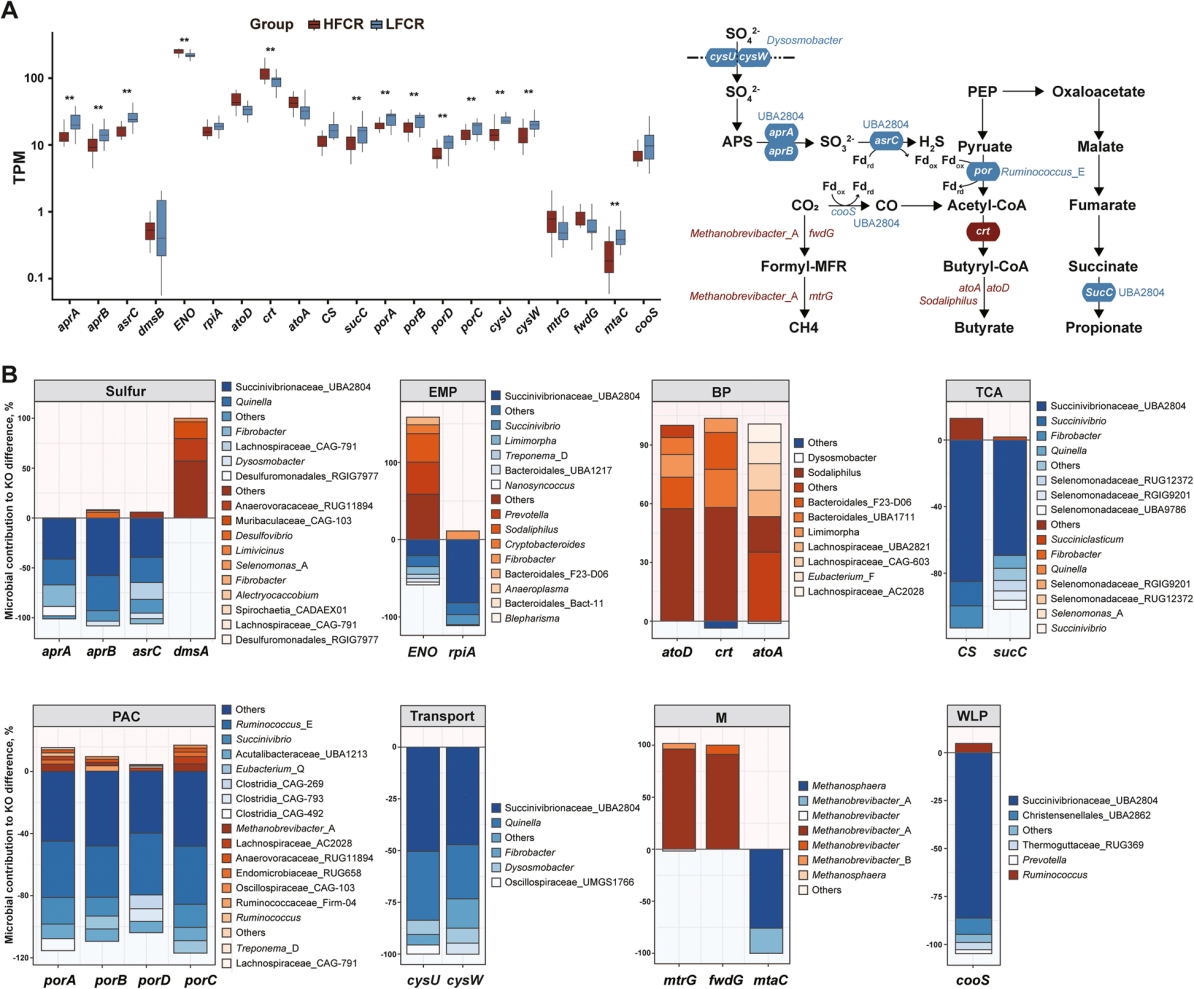
Differential pathways related to carbohydrate fermentation and hydrogen metabolism between HFCR and LFCR groups. **A** Boxplots of differentially abundant KOs, including sulfur reduction (*aprA*, *aprB*, *asrC*, *dmsA*), glycolysis (*ENO*, *rpiA*), butyrate synthesis (*crt*, *atoA*, *atoD*), TCA cycle (*CS*, *sucC*), pyruvate oxidation (*porA–D*), transporters (*cycU*, *cysW*), methanogenesis (*fwdG*, *mtrG*, *mtaC*), and the Wood–Ljungdahl pathway (*cooS*). Asterisks denote significant differences (^*^P < 0.05; ^**^P < 0.01), while genes without asterisks exhibited trends (0.05 ≤ *P* < 0.10). The schematic (right) highlights the positions of selected KOs within major fermentation routes. PEP, phosphoenolpyruvate; APS, adenosine-5'-phosphosulfate; Fd, ferredoxin (Fdᵣ_d_/Fdₒₓ, reduced/oxidized). **B** Microbial contributions to KO-level differences across representative pathways. Stacked bar plots indicate the proportional contribution of microbial genera to the observed KO differences, plotted using the same approach as in Fig. 4. Sulfur, sulfidogenesis; EMP, embden-meyerhof-parnas; BP, butyrate production; TCA, tricarboxylic acid cycle; PAC, pyruvate to acetyl-CoA; Transport, sulfate/thiosulfate transport; M, methanogenesis; WLP, Wood–Ljungdahl pathway

At the KO level, LFCR showed significantly higher abundances of pyruvate:ferredoxin oxidoreductase (*porA–D*), succinate synthetase (*sucC*), and methyltransferase for methylotrophic methanogenesis (*mtaC*) (*P* < 0.05; Fig. 6A). Citrate synthase (*CS*) and acetyl-CoA synthase (*cooS*) also tended to be higher in LFCR (0.05 ≤ *P* < 0.10). LFCR exhibited lower abundances of glycolytic enzymes *ENO*, butyrate synthesis genes (*crt*, *P* < 0.05; *atoA*, *atoD, *0.05 ≤ *P <* 0.10), and hydrogenotrophic methanogenesis genes (*fwdG*, *mtrG*, 0.05 ≤ *P* < 0.10). Taxonomically, *porA–D* derived mainly from *Ruminococcus*_E, *sucC* and *CS* were from Succinivibrionaceae_UBA2804 (*P* = 0.054); and butyrate synthesis genes from *Sodaliphilus* (*P* < 0.05), which was significantly enriched in HFCR (Fig. 6B).

Hydrogen (H_2_) serves as a central intermediate in these anaerobic networks, and a total of 3,686 non-redundant hydrogenase genes were identified (Additional file 1: Table S7). The majority belonged to [FeFe]-hydrogenases (95.2%), with [FeFe] Group A1 alone accounting for 58.65% of all sequences, followed by Group A3 (17.20%) and Group B (9.44%). HydDB-based classification divided these into six categories: fermentative, electron-bifurcating, sensory, methanogenic, respiratory, and energy-converting (*P* > 0.05, Additional file 2: Fig. S3A).

Terminal reductases associated with hydrogen utilization showed group-dependent but largely non-significant patterns. Genes involved in sulfate reduction (*aprA*, *aprB*, *asrC*) and transport (*cysU*, *cysW*) were significantly enriched in LFCR (*P* < 0.05), with key contributions from Succinivibrionaceae_UBA2804, *Quinella *and* Dysosmobacter* (Fig. 6B). Genes involved in nitrate ammonification and denitrification (*narG*, *narH*, *narI*, *nosZ*, *napA*, *nrfA*, *nrfH*) did not differ between HFCR and LFCR (*P* > 0.05; Additional file 2: Fig. S3C).

### Tissue-specific transcriptional programs associated with feed efficiency

**Fig. 7:**
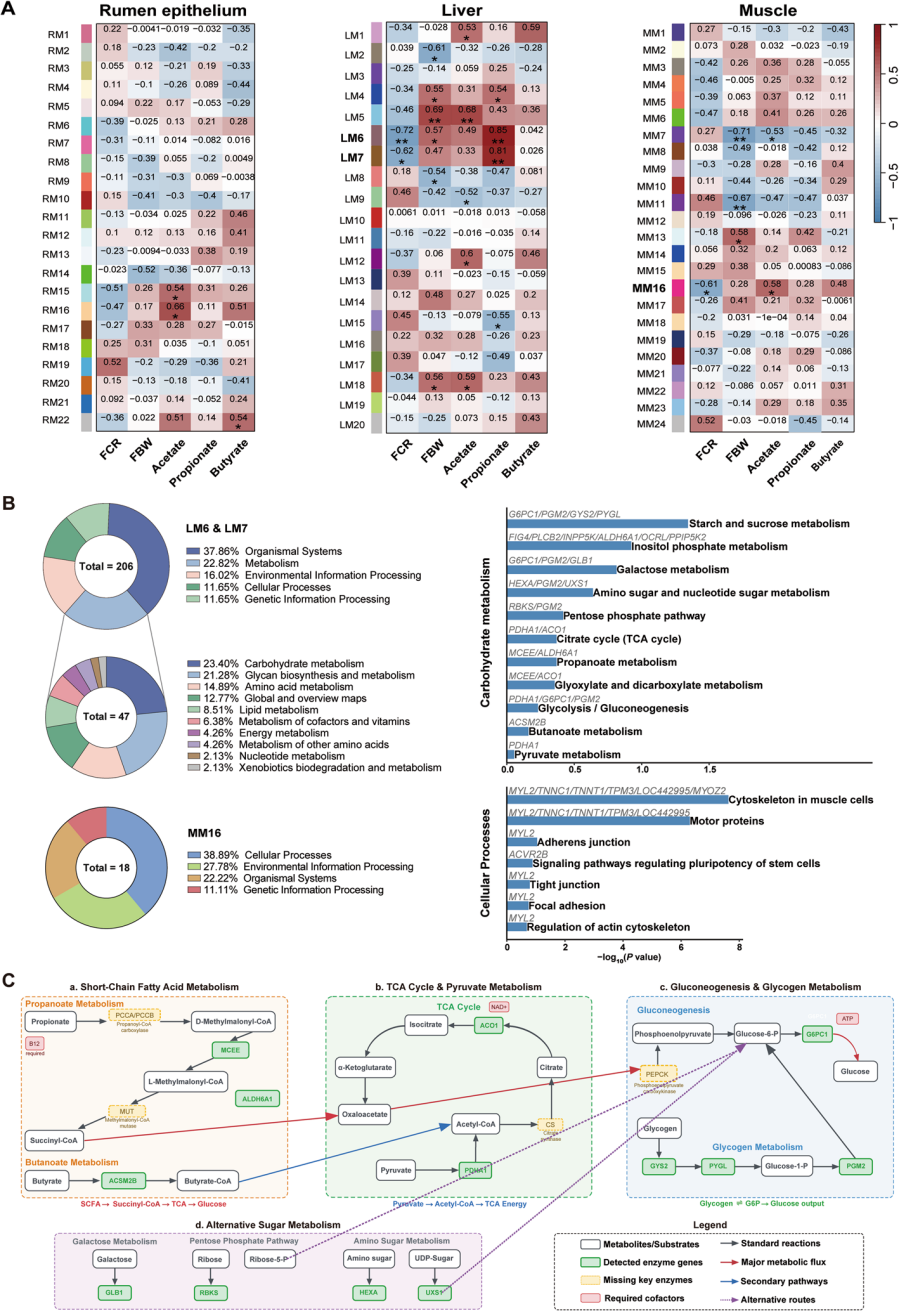
Cross-tissue module–trait correlations, KEGG functional distribution, and integrated hepatic carbohydrate flux. **A** WGCNA module–trait correlations with FCR across rumen epithelium, liver, and muscle. Significant associations were observed for liver M6/M7 and muscle M16 (*P* < 0.05) *P < 0.05, **P < 0.01. **B** KEGG enrichment of liver modules, with metabolism as the dominant level 1 category and carbohydrate metabolism as the leading level 2 category; enriched level 3 pathways are shown on the right. **C** KEGG-based metabolism-only schematic of hepatic flux. Solid edges/bold labels indicate genes detected in our modules; dashed/italic denote mandatory but non-detected steps

RNA-seq generated a total of 906,346,909 clean reads across rumen epithelium, liver, and muscle tissues, with > 95% Q30 bases and > 90% mapping rates [24]. After filtering (FPKM ≤ 1), 11,292 genes in rumen epithelium, 9,246 in liver, and 9,669 in muscle were retained. WGCNA revealed tissue-specific associations with FCR: two liver modules (LM6, LM7) and one muscle module (MM16) correlated significantly with FCR (*P* < 0.05), whereas no associations were detected in the rumen epithelium (Fig. 7A).

KEGG enrichment of these modules revealed a striking contrast between liver and muscle. The liver modules were enriched in metabolic pathways (KEGG level 1), with carbohydrate metabolism as the largest category, followed by glycan biosynthesis, amino acid metabolism, and lipid metabolism. At KEGG level 3, enriched pathways included glycolysis/gluconeogenesis, the TCA cycle, pyruvate metabolism, propanoate metabolism, and starch and sucrose metabolism, with enzymes such as *CS*, *PKM*, and *PDHA1* detected in FCR-associated modules (Fig. 7B). In muscle, MM16 was enriched for structural and cytoskeletal pathways, including regulation of actin cytoskeleton, focal adhesion, adherens junction, tight junction, and signaling pathways regulating pluripotency of stem cells. Hub genes included *MYL2*, *TNNC1*, *TNNT1*, *TPM3*, *MYOZ2*, and *ACVR2B*. No carbohydrate metabolism pathways were enriched in MM16.

Liver modules contained 15 enzyme genes spanning 11 metabolic pathways (Fig. 7B and C): glycogen metabolism (*GYS2*, *PYGL*, *PGM2*), glucose export (*G6PC1*), SCFA-linking enzymes (*MCEE*, *ALDH6A1*, *ACSM2B*), TCA cycle enzymes (*PDHA1*, *ACO1*), galactose metabolism (*GLB1*), pentose phosphate pathway (*RBKS*), amino sugar metabolism (*HEXA*, *UXS1*), and inositol phosphate metabolism (FIG4, *PLCB2*, *INPP5K*, *OCRL*, *PPIP5K2*). Hub genes *G6PC1* and *PGM2* connected multiple pathways (Fig. 7C). Further analysis of the relationships between microbial functional genes and host modules showed that fermentation-pathway genes (*porA*, *porB*, *porC*, *cooS*, *sucC*) were positively correlated with hepatic modules, whereas methanogenesis-related genes (*mtrG*) exhibited inverse correlations with muscular modules. Sulfate-reduction genes (*aprA*, *aprB*, *asrC*, *cysU*, *cysW*) were positively associated with hepatic modules, and the amino acid biosynthesis gene *lysC* also correlated with liver modules (Additional file 2: Fig. S5).

## Discussion

Feed efficiency in ruminants is a multifactorial trait shaped by the interplay of microbial fermentation, nutrient absorption, and host metabolism. In our study, animals with LFCR achieved superior growth performance without greater feed intake, underscoring that efficiency is primarily determined by nutrient utilization rather than feed consumption. This agrees with previous cattle and sheep studies showing that FCR variation cannot be explained by intake alone [18, 50]. Elevated ruminal propionate in the LFCR group highlights a glucogenic orientation, as propionate serves as the major gluconeogenic precursor in ruminants. Prior work similarly linked higher propionate and lower acetate:propionate ratios with improved energy capture and reduced methane emissions [7, 51]. Notably, total VFA concentration did not differ between groups, suggesting that feed efficiency reflects redistribution of carbon flux rather than overall fermentation intensity [52]. This highlights propionate enrichment as a core metabolic hallmark of efficient ruminants.

Despite marked phenotypic divergence, microbial diversity (α- and β-diversity) remained stable, indicating that efficiency differences arise from compositional and functional shifts in specific taxa rather than community richness [18, 53, 54]. LFCR associated with hemicellulose degradation and propionate production [17, 55, 56]. These features align with the elevated propionate phenotype and suggest more efficient carbohydrate turnover. Conversely, HFCR animals were enriched in *Sodaliphilus* and Bacilli_UBA2450, which possess limited glycolytic capacity and depend on syntrophic interactions [11, 57]. Such compositional contrasts likely contribute to differential energy yield from carbohydrate fermentation, consistent with the notion that feed efficiency is governed by the presence of keystone taxa rather than overall diversity [54].

Functional profiling revealed distinct metabolic orientations between groups. LFCR animals exhibited enrichment in amino acid biosynthesis, central carbon metabolism, and sulfur metabolism, whereas HFCR animals were enriched in ribosomal and lipid metabolism. Amino acid pathways—particularly those related to branched-chain, sulfur-containing, aromatic, and histidine biosynthesis—accounted for a major portion of LFCR-enriched functions and co-occurred with the TCA cycle, propionate metabolism, and carbon fixation [58]. This convergence suggests a coordinated carbon–nitrogen coupling strategy, allowing efficient microbiomes to redirect carbohydrate-derived carbon skeletons toward amino acid synthesis and microbial protein generation, thereby improving nitrogen utilization efficiency [59, 60]. In contrast, the dominance of ribosomal pathways in HFCR animals may represent an energetically costly growth-oriented strategy emphasizing microbial biomass turnover rather than nutrient provision to the host [61].

Although the total abundance of CAZyme classes was conserved, family-level analysis revealed distinct carbohydrate utilization strategies. LFCR animals were enriched in EPS-glycosyltransferases (GT39, GT7), starch-active GH126, and hemicellulose-binding CBM27, reflecting a microbiome optimized for soluble carbohydrate and extracellular polysaccharide turnover [62–65]. Conversely, inefficient animals displayed greater capacity for degrading host glycans and structural carbohydrates (e.g., GH89, CE14, CBM8, AA10) [66–68]. These patterns indicate that inefficient microbiomes rely more on recalcitrant or host-derived substrates, which may offer flexibility under nutrient limitation but incur higher metabolic costs and slower energy release [68].

Downstream fermentation and hydrogen metabolism provided further evidence of divergent energy conservation [69, 70]. LFCR animals exhibited higher abundances of *porA–D* and *sucC*, consistent with succinate–propionate pathways that maximize ATP yield and minimize hydrogen accumulation [71, 72]. Succinivibrionaceae_UBA2804 contributed to succinate and sulfate reduction, indicating flexible electron disposal routes [17]. In contrast, the metabolic configuration of HFCR animals reflected a hydrogen economy biased toward butyrate biosynthesis and hydrogenotrophic methanogenesis, thereby limiting the routing of reductive equivalents toward propionate formation [73]. The enrichment of sulfate reduction genes (*aprA*, *aprB*, *asrC*, *cysU*/*W*) in LFCR provides an additional alternative hydrogen sink, potentially reducing methane emissions while maintaining redox balance [74]. At the same time, pathway patterns revealed a clear divergence between methylotrophic and hydrogenotrophic methanogenesis: *mtaC* (methylotrophic) was maintained or slightly elevated in LFCR, whereas hydrogenotrophic markers (*mcrA*, *fwdG*, *mtrG*) were reduced. These trends indicate that hydrogen flow in LFCR animals was redirected from hydrogenotrophic methanogenesis toward fumarate and sulfate reduction, supporting alternative electron-consuming pathways [66]. Such reallocation of reducing equivalents reflects an “electron-efficient” microbiome that coordinates carbon and hydrogen metabolism to enhance energy conservation and feed utilization [67].

Transcriptomic analysis revealed tissue-specific regulatory programs that complement microbial outputs. In the liver, two FCR-associated modules (M6, M7) were enriched in carbohydrate metabolism, encompassing glycolysis/gluconeogenesis, the TCA cycle, propanoate metabolism, and glycogen turnover [75]. Key genes (*GYS2*, *PYGL*, *PGM2*, *G6PC1*) formed a complete glycogen cycling and glucose export system, while *MCEE*, *ALDH6A1*, and *ACSM2B* linked microbial SCFAs to central carbon metabolism [76, 77]. These findings identify glucose-6-phosphate as a metabolic hub for integrating microbial propionate with hepatic gluconeogenesis. In contrast, the muscle module (M16) was dominated by structural and contractile genes (*MYL2*, *TNNC1*, *TNNT1*, *TPM3*, *MYOZ2, ACVR2B*), indicating that skeletal muscle contributes to efficiency through optimizing ATP utilization in contraction rather than direct metabolic flux [78, 79]. However, no FCR-associated modules were identified in the rumen epithelium. This may be explained by its physiological role: amino acids synthesized in the rumen are not absorbed there but pass into the small intestine, while propionate is primarily metabolized in the liver rather than the epithelium [80, 81]. These constraints likely mask transcriptomic differences despite the epithelium’s essential role in VFA absorption and barrier function [82, 83]. This observation underscores the need to interpret rumen function in the context of systemic host–microbiome integration rather than in isolation.

Collectively, our findings reveal that high-efficiency ruminants orchestrate a tightly integrated microbiota–host axis. On the microbial side, LFCR animals displayed functional convergence on amino acid biosynthesis, propionate-oriented fermentation, hydrogen redirection into alternative sinks, and CAZymes specialized for soluble carbohydrates [84]. On the host side, the liver was transcriptionally reprogrammed toward glucose homeostasis and glycogen turnover, while skeletal muscle optimized energy expenditure through contractile efficiency. These cross-kingdom adaptations collectively establish a metabolic continuum from rumen fermentation through hepatic integration to muscle utilization, minimizing energy loss and maximizing nutrient capture [85, 86]. This framework emphasizes that feed efficiency arises from coordinated microbial–host interactions rather than single components, providing a conceptual model to guide dietary, microbial, and genetic interventions for sustainable ruminant production. While this study provides an integrated view of the microbiota–host axis, the taxonomic resolution for eukaryotes remains limited because the analysis was based on gene-level profiles optimized for prokaryotes. Consequently, the reported taxonomic results should be interpreted as representing the functional gene-encoding fraction of the microbiome, rather than the entire microbial community. In addition, as only male Hu sheep were analyzed, further investigations in females are warranted to evaluate potential sex-dependent microbial and metabolic responses.

## Conclusion

Feed conversion efficiency in Hu sheep is not determined by feed intake or global microbial diversity but by how microbial functions and host metabolism are coordinated. Efficient animals exhibited a rumen microbiome biased toward propionate production and amino acid metabolism, coupled with hepatic and muscular programs that optimized nutrient conversion and energy use. These results provide mechanistic insight into the microbiota–host interplay underlying feed efficiency and point to microbial and host targets for improving ruminant productivity.

## Supplementary Information

Additional file 1: Table S1. Shotgun metagenome sequencing yield and quality metrics per sample. Table S2. Quality-filtered clean reads statistics for rumen metagenomes. Table S3. Summary of host and feed read removal from rumen metagenomic datasets. Table S4. Per-sample de novo assembly statistics. Table S5. Domain-level taxonomic composition of rumen metagenomes. Table S6. Summary of CAZyme gene catalog and classification. Table S7. Classification of hydrogenase genes identified in HFCR and LFCR sheep.

Additional file 2: Fig. S1. Comprehensive KEGG pathway landscape from differential KOs. Fig. S2. Class-level profiles of CAZymes in HFCR and LFCR groups. Fig. S3. Terminal reductases and hydrogenase subclasses across HFCR and LFCR groups. Fig. S4. Genus-resolved heatmap of terminal reductases and hydrogenase subclasses in HFCR and LFCR rumen metagenomes. Fig. S5. Correlation heatmap between microbial functional genes and host co-expression modules. Fig. S6. Validation of hepatic gene expression by RT-qPCR.

## Data Availability

The raw metagenomic sequencing data generated in this study have been deposited in the NCBI Sequence Read Archive (SRA) under BioProject accession number PRJNA1309569 and are publicly accessible. The raw RNA-seq datasets are available under BioProject accession number PRJNA1226734 and are already publicly accessible. These datasets are part of a larger multi-omics project and have also been used in related manuscripts that are currently under peer review.

## Abbreviations

ADFI: Average daily feed intake
ADG: Average daily gain
DMI: Dry matter intake
FCR: Feed conversion ratio
FE: Feed efficiency
FPKM: Fragments per kilobase of transcript per million mapped reads
HFCR: High feed-conversion ratio
KEGG: Kyoto Encyclopedia of Genes and Genomes
KO: KEGG Orthology
LFCR: Low feed-conversion ratio
PCoA: Principal coordinates analysis
RFI: Residual feed intake
TOM: Topological overlap matrix
VFA: Volatile fatty acid
WGCNA: Weighted gene co-expression network analysis

## References

[1] Du Y, Ge Y, Chang J. Global strategies to minimize environmental impacts of ruminant production. Annu Rev Anim Biosci. 2022;10:227–40. 10.1146/annurev-animal-020420-043152. 34780247 10.1146/annurev-animal-020420-043152

[2] Frazier AN, Beck MR, Waldrip H, Koziel JA. Connecting the ruminant microbiome to climate change: insights from current ecological and evolutionary concepts. Front Microbiol. 2024;15:1503315. 10.3389/fmicb.2024.1503315. 39687868 10.3389/fmicb.2024.1503315 PMC11646987

[3] Tan R, Zhou M, Li F, Guan L. Identifying active rumen epithelial associated bacteria and archaea in beef cattle divergent in feed efficiency using total RNA-seq. Curr Res Microb Sci. 2021;2:100064. 10.1016/j.crmicr.2021.100064. 34841354 10.1016/j.crmicr.2021.100064 PMC8610342

[4] Badhan A, Wang Y, Terry S, Gruninger R, Guan LL, McAllister TA. Interplay of rumen microbiome and the cattle host in modulating feed efficiency and methane emissions. J Dairy Sci. 2025;108:5489–501. 10.3168/jds.2024-26063. 40221043 10.3168/jds.2024-26063

[5] Li F, Li C, Chen Y, Liu J, Zhang C, Irving B, et al. Host genetics influence the rumen microbiota and heritable rumen microbial features associate with feed efficiency in cattle. Microbiome. 2019;7:92. 10.1186/s40168-019-0699-1. 31196178 10.1186/s40168-019-0699-1 PMC6567441

[6] Li F, Hitch TCA, Chen Y, Creevey CJ, Guan LL. Comparative metagenomic and metatranscriptomic analyses reveal the breed effect on the rumen microbiome and its associations with feed efficiency in beef cattle. Microbiome. 2019;7:6. 10.1186/s40168-019-0618-5. 30642389 10.1186/s40168-019-0618-5 PMC6332916

[7] Zhou G, Li J, Liang X, Yang B, He X, Tang H, et al. Multi-omics revealed the mechanism of feed efficiency in sheep by the combined action of the host and rumen microbiota. Anim Nutr. 2024;18:367–79. 10.1016/j.aninu.2024.04.009. 39290858 10.1016/j.aninu.2024.04.009 PMC11406083

[8] Auffret MD, Stewart RD, Dewhurst RJ, Duthie C-A, Watson M, Roehe R. Identification of microbial genetic capacities and potential mechanisms within the rumen microbiome explaining differences in beef cattle feed efficiency. Front Microbiol. 2020;11:1229. 10.3389/fmicb.2020.01229. 32582125 10.3389/fmicb.2020.01229 PMC7292206

[9] Wang W, Zhang Y, Zhang X, Li C, Yuan L, Zhang D, et al. Heritability and recursive influence of host genetics on the rumen microbiota drive body weight variance in male Hu sheep lambs. Microbiome. 2023;11:197. 10.1186/s40168-023-01642-7. 37644504 10.1186/s40168-023-01642-7 PMC10463499

[10] Hagen L, Brooke C, Shaw C, Norbeck A, Piao H, Arntzen M, et al. Proteome specialization of anaerobic fungi during ruminal degradation of recalcitrant plant fiber. ISME J. 2021;15:421–34. 10.1038/s41396-020-00769-x. 32929206 10.1038/s41396-020-00769-x PMC8026616

[11] Lin L, Ma H, Zhang J, Yang H, Zhang J, Lai Z, et al. Lignocellulolytic microbiomes orchestrating degradation cascades in the rumen of dairy cattle and their diet-influenced key degradation phases. Anim Adv. 2024;1:e002. 10.48130/animadv-0024-0002.

[12] Liang J, Fang W, Chang J, Zhang G, Ma W, Nabi M, et al. Long-term rumen microorganism fermentation of corn stover in vitro for volatile fatty acid production. Bioresour Technol. 2022;358:127447. 10.1016/j.biortech.2022.127447. 35690238 10.1016/j.biortech.2022.127447

[13] Wardman J, Bains R, Rahfeld P, Withers S. Carbohydrate-active enzymes (CAZymes) in the gut microbiome. Nat Rev Microbiol. 2022;20:542–56. 10.1038/s41579-022-00712-1. 35347288 10.1038/s41579-022-00712-1

[14] Fonoll X, Zhu K, Aley L, Shrestha S, Raskin L. Simulating rumen conditions using an anaerobic dynamic membrane bioreactor to enhance hydrolysis of lignocellulosic biomass. Environ Sci Technol. 2024;58:1741–51. 10.1021/acs.est.3c06478. 38184844 10.1021/acs.est.3c06478

[15] Bergman E. Energy contributions of volatile fatty acids from the gastrointestinal tract in various species. Physiol Rev. 1990;70:567–90. 10.1152/physrev.1990.70.2.567. 2181501 10.1152/physrev.1990.70.2.567

[16] Shi H, Shi Q, Grodner B, Lenz J, Zipfel W, Brito I, et al. Highly multiplexed spatial mapping of microbial communities. Nature. 2020;588:676–81. 10.1038/s41586-020-2983-4. 33268897 10.1038/s41586-020-2983-4 PMC8050837

[17] Jia M, Zhu S, Xue M, Chen H, Xu J, Song M, et al. Single-cell transcriptomics across 2,534 microbial species reveals functional heterogeneity in the rumen microbiome. Nat Microbiol. 2024;9:1884–981. 10.1038/s41564-024-01723-9. 38866938 10.1038/s41564-024-01723-9

[18] Xue M, Xie Y, Zhong Y, Ma X, Sun H, Liu J. Integrated meta-omics reveals new ruminal microbial features associated with feed efficiency in dairy cattle. Microbiome. 2022;10:32. 10.1186/s40168-022-01228-9. 35172905 10.1186/s40168-022-01228-9 PMC8849036

[19] Xue M, Xie Y, Zang X, Zhong Y, Ma X, Sun H, et al. Deciphering functional groups of rumen microbiome and their underlying potentially causal relationships in shaping host traits. iMeta. 2024;3:e225. 10.1002/imt2.225. 39135684 10.1002/imt2.225 PMC11316931

[20] Fonseca P, Lam S, Chen Y, Waters S, Guan L, Cánovas A. Multi-breed host rumen epithelium transcriptome and microbiome associations and their relationship with beef cattle feed efficiency. Sci Rep. 2023;13:16209. 10.1038/s41598-023-43097-8. 37758745 10.1038/s41598-023-43097-8 PMC10533831

[21] Chen J, Zhang X, Chang X, Wei B, Fang Y, Song S, et al. Multi-omics analysis reveals the effects of host-rumen microbiota interactions on growth performance in a goat model. Front Microbiol. 2024;15:1445223. 10.3389/fmicb.2024.1445223. 39314883 10.3389/fmicb.2024.1445223 PMC11417024

[22] Zhang D, Cheng J, Li X, Huang K, Yuan L, Zhao Y, et al. Comprehensive multi‐tissue epigenome atlas in sheep: a resource for complex traits, domestication, and breeding. iMeta. 2024;3:e254. 10.1002/imt2.25. 10.1002/imt2.254 PMC11683475 39742295

[23] Ministry of Agriculture and Rural Affairs of the People’s Republic of China. Nutrient requirements of meat-type sheep and goat: NY/T 816–2021. Beijing: China Agriculture Press; 2021.

[24] Jia X, Li J, Zhang Y, Tian B, Mao S, Liu J, et al. Transcriptomic profiling of rumen epithelium, liver, and muscle reveals tissue-specific gene expression patterns in Hu sheep. BMC Genomics. 2025;26:12311. 10.1186/s12864-025-12311-4. 10.1186/s12864-025-12311-4 PMC12729863 41257554

[25] Sun D, Yin Y, Guo C, Liu L, Mao S, Zhu W, et al. Transcriptomic analysis reveals the molecular mechanisms of rumen wall morphological and functional development induced by different solid diet introduction in a lamb model. J Anim Sci Biotechnol. 2021;12:33. 10.1186/s40104-021-00556-4. 33750470 10.1186/s40104-021-00556-4 PMC7944623

[26] Yu Z, Morrison M. Improved extraction of PCR-quality community DNA from digesta and fecal samples. Biotechniques. 2004;36:808–12. 10.2144/04365ST04. 15152600 10.2144/04365ST04

[27] Bolger A, Lohse M, Usadel B. Trimmomatic: a flexible trimmer for Illumina sequence data. Bioinformatics. 2014;30:2114–20. 10.1093/bioinformatics/btu170. 24695404 10.1093/bioinformatics/btu170 PMC4103590

[28] Li H, Durbin R. Fast and accurate long-read alignment with Burrows-Wheeler transform. Bioinformatics. 2010;26:589–95. 10.1093/bioinformatics/btp698. 20080505 10.1093/bioinformatics/btp698 PMC2828108

[29] Li D, Liu C, Luo R, Sadakane K, Lam T. MEGAHIT: an ultra-fast single-node solution for large and complex metagenomics assembly via succinct de Bruijn graph. Bioinformatics. 2015;31:1674–6. 10.1093/bioinformatics/btv033. 25609793 10.1093/bioinformatics/btv033

[30] Hyatt D, Chen G, Locascio P, Land M, Larimer F, Hauser L. Prodigal: prokaryotic gene recognition and translation initiation site identification. BMC Bioinformatics. 2010;11:119. 10.1186/1471-2105-11-119. 20211023 10.1186/1471-2105-11-119 PMC2848648

[31] Fu L, Niu B, Zhu Z, Wu S, Li W. CD-HIT: accelerated for clustering the next-generation sequencing data. Bioinformatics. 2012;28:3150–2. 10.1093/bioinformatics/bts565. 23060610 10.1093/bioinformatics/bts565 PMC3516142

[32] Li J, Jia H, Cai X, Zhong H, Feng Q, Sunagawa S, et al. An integrated catalog of reference genes in the human gut microbiome. Nat Biotechnol. 2014;32:834–41. 10.1038/nbt.2942. 24997786 10.1038/nbt.2942

[33] Madeira F, Pearce M, Tivey A, Basutkar P, Lee J, Edbali O, et al. Search and sequence analysis tools services from EMBL-EBI in 2022. Nucleic Acids Res. 2022;50:W276–9. 10.1093/nar/gkac240. 35412617 10.1093/nar/gkac240 PMC9252731

[34] Kanehisa M, Goto S, Kawashima S, Okuno Y, Hattori M. The KEGG resource for deciphering the genome. Nucleic Acids Res. 2004;32:D277–80. 10.1093/nar/gkh063. 14681412 10.1093/nar/gkh063 PMC308797

[35] Buchfink B, Xie C, Huson D. Fast and sensitive protein alignment using DIAMOND. Nat Methods. 2015;12:59–60. 10.1038/nmeth.3176. 25402007 10.1038/nmeth.3176

[36] Lombard V, Golaconda Ramulu H, Drula E, Coutinho P, Henrissat B. The carbohydrate-active enzymes database (CAZy) in 2013. Nucleic Acids Res. 2014;42:D490–5. 10.1093/nar/gkt117. 24270786 10.1093/nar/gkt1178 PMC3965031

[37] Potter S, Luciani A, Eddy R, Park Y, Lopez R, Finn R. HMMER web server: 2018 update. Nucleic Acids Res. 2018;46:W200–4. 10.1093/nar/gky448. 29905871 10.1093/nar/gky448 PMC6030962

[38] Søndergaard D, Pedersen C, Greening C. HydDB: a web tool for hydrogenase classification and analysis. Sci Rep. 2016;6:34212. 10.1038/srep34212. 27670643 10.1038/srep34212 PMC5037454

[39] Wagner G, Kin K, Lynch V. Measurement of mRNA abundance using RNA-seq data: RPKM measure is inconsistent among samples. Theory Biosci. 2012;131:281–5. 10.1007/s12064-012-0162-3. 22872506 10.1007/s12064-012-0162-3

[40] Kim D, Langmead B, Salzberg S. HISAT: a fast spliced aligner with low memory requirements. Nat Methods. 2015;12:357–60. 10.1038/nmeth.3317. 25751142 10.1038/nmeth.3317 PMC4655817

[41] Pertea M, Pertea G, Antonescu C, Chang T, Mendell J, Salzberg S. Stringtie enables improved reconstruction of a transcriptome from RNA-seq reads. Nat Biotechnol. 2015;33:290–5. 10.1038/nbt.3122. 25690850 10.1038/nbt.3122 PMC4643835

[42] Wu T, Hu E, Xu S, Chen M, Guo P, Dai Z, et al. Clusterprofiler 4.0: a universal enrichment tool for interpreting omics data. Innov (Camb). 2021;2:100141. 10.1016/j.xinn.2021.100141. 10.1016/j.xinn.2021.100141 PMC8454663 34557778

[43] Greenfest-Allen E, Cartailler J, Magnuson M, Stoeckert C. iterativeWGCNA: iterative refinement to improve module detection from WGCNA co-expression networks. bioRxiv. 2017:234062. 10.1101/234062.

[44] Dixon P. VEGAN, a package of R functions for community ecology. J Veg Sci. 2003;14:927–30. 10.1111/j.1654-1103.2003.tb02228.x.

[45] Kassambara A. ggpubr: “ggplot2” based publication ready plots. https://cran.r-project.org/web/packages/ggpubr/index.html.

[46] Kolde R. pheatmap: pretty heatmaps. https://cran.r-project.org/web/packages/pheatmap/index.html.

[47] Auguie B, Antonov A. gridExtra: miscellaneous functions for “Grid” graphics. 2017. https://cran.r-project.org/web/packages/gridExtra/index.html.

[48] Xiao N. ggsci: scientific journal and sci-fi themed color palettes for “ggplot2”. 2025. https://cran.r-project.org/web/packages/ggsci/index.html.

[49] Auguie B. egg: extensions for “ggplot2”: custom geom, custom themes, plot alignment, labelled panels, symmetric scales, and fixed panel size. 2019. https://cran.r-project.org/web/packages/egg/index.html.

[50] Giráldez F, Santos N, Santos A, Valdés C, López S, Andrés S. Fattening lambs with divergent residual feed intakes and weight gains: unravelling mechanisms driving feed efficiency. Anim Feed Sci Technol. 2021;273:114821. 10.1016/j.anifeedsci.2021.114821.

[51] Wallace R, Sasson G, Garnsworthy P, Tapio I, Gregson E, Bani P, et al. A heritable subset of the core rumen microbiome dictates dairy cow productivity and emissions. Sci Adv. 2019;5:eaav8391. 10.1126/sciadv.aav8391. 31281883 10.1126/sciadv.aav8391 PMC6609165

[52] Strachan C, Yu X, Neubauer V, Mueller A, Wagner M, Zebeli Q, et al. Differential carbon utilization enables co-existence of recently speciated Campylobacteraceae in the cow rumen epithelial microbiome. Nat Microbiol. 2023;8:309–20. 10.1038/s41564-022-01300-y. 36635570 10.1038/s41564-022-01300-y PMC9894753

[53] Clemmons B, Martino C, Powers J, Campagna S, Voy B, Donohoe D, et al. Rumen bacteria and serum metabolites predictive of feed efficiency phenotypes in beef cattle. Sci Rep. 2019;9:19265. 10.1038/s41598-019-55978-y. 31848455 10.1038/s41598-019-55978-y PMC6917770

[54] Banerjee S, Schlaeppi K, van der Heijden MGA. Keystone taxa as drivers of microbiome structure and functioning. Nat Rev Microbiol. 2018;16:567–76. 10.1038/s41579-018-0024-1. 29789680 10.1038/s41579-018-0024-1

[55] Hu W, Wu Y, Bian Y, Zheng X, Chen Y, Dong L. Joint effects of carbon nanotubes and copper oxide nanoparticles on fermentation metabolism towards *Saccharofermentans acetigenes*: enhancing environmental adaptability and transcriptional expression. Bioresour Technol. 2021;336:125318. 10.1016/j.biortech.2021.125318. 34049169 10.1016/j.biortech.2021.125318

[56] Kong F, Wang S, Zhang Y, Li C, Dai D, Wang Y, et al. Alanine derived from *Ruminococcus_*E *bovis* alleviates energy metabolic disorders during the peripartum period by providing glucogenic precursors. Research. 2025;8:0682. 10.34133/research.0682. 10.34133/research.0682 PMC12022398 40290137

[57] Holman D, Kommadath A, Tingley J, Abbott D. Novel insights into the pig gut microbiome using metagenome-assembled genomes. Microbiol Spectr. 2022;10:e02380–22. 10.1128/spectrum.02380-22. 35880887 10.1128/spectrum.02380-22 PMC9431278

[58] Xue M, Xie Y, Zhong Y, Liu J, Guan L, Sun H. Ruminal resistome of dairy cattle is individualized and the resistotypes are associated with milking traits. Anim Microbiome. 2021;3:18. 10.1186/s42523-021-00081-9. 33568223 10.1186/s42523-021-00081-9 PMC7877042

[59] Sasson G, Moraïs S, Kokou F, Plate K, Trautwein-Schult A, Jami E, et al. Metaproteome plasticity sheds light on the ecology of the rumen microbiome and its connection to host traits. ISME J. 2022;16:2610–21. 10.1038/s41396-022-01295-8. 35974086 10.1038/s41396-022-01295-8 PMC9563048

[60] Liu B, Sträuber H, Centler F, Harms H, Rocha U, Kleinsteuber S. Functional redundancy secures resilience of chain elongation communities upon pH shifts in closed bioreactor ecosystems. Environ Sci Technol. 2023;57:12501–13. 10.1021/acs.est.2c09573. 37097211 10.1021/acs.est.2c09573 PMC10666546

[61] Daisley B, Koenig D, Engelbrecht K, Doney L, Hards K, Al K, et al. Emerging connections between gut microbiome bioenergetics and chronic metabolic diseases. Cell Rep. 2021;37:110087. 10.1016/j.celrep.2021.110087. 34879270 10.1016/j.celrep.2021.110087

[62] Jacob M, Inka B. Biosynthesis of bacterial polysaccharides. Adv Carbohydr Chem Biochem. 2021;78:143–78. 10.1016/B978-0-12-819475-1.00097-3.

[63] Kelly S, Williams D, Nothof J, Kim T, Lowary T, Kimber M, et al. The biosynthetic origin of ribofuranose in bacterial polysaccharides. Nat Chem Biol. 2022;18:530–7. 10.1038/s41589-022-01006-6. 35393575 10.1038/s41589-022-01006-6

[64] Hodorova M, Janeček Š. The family GH126 – its relatedness to and differentiation from GH8 and GH48 including the intermediary sequences. Food Biosci. 2024;62:105064. 10.1016/j.fbio.2024.105064.

[65] Yu Q, Yang J, Liu L, Huang Y, Wang E, Li D, et al. One-step immobilization of chitosanase on microcrystalline cellulose using a carbohydrate binding module family 2. Carbohydr Polym. 2025;353:123291. 10.1016/j.carbpol.2025.123291. 39914986 10.1016/j.carbpol.2025.123291

[66] Liu N, Yu W, Guo X, Chen J, Xia D, Yu J, et al. Oxidative cleavage of cellulose in the horse gut. Microb Cell Fact. 2022;21:38. 10.1186/s12934-022-01767-8. 35279161 10.1186/s12934-022-01767-8 PMC8917663

[67] Liberato M, Campos B, Tomazetto G, Crouch I, Garcia W, Zeri A, et al. Unique properties of a *Dictyostelium discoideum* carbohydrate-binding module expand our understanding of CBM-ligand interactions. J Biol Chem. 2022;298:101891. 10.1016/j.jbc.2022.101891. 35378128 10.1016/j.jbc.2022.101891 PMC9079177

[68] Gao Y, Zhang Y, Han Z, Wang C, Zhang L, Qiu J. Two mixed-valent cerium oxo clusters: synthesis, structure, and self-assembly. Front Chem. 2024;12:1507834. 10.3389/fchem.2024.1507834. 39686981 10.3389/fchem.2024.1507834 PMC11646716

[69] Peng X, Wilken S, Lankiewicz T, Gilmore S, Brown J, Henske J, et al. Genomic and functional analyses of fungal and bacterial consortia that enable lignocellulose breakdown in goat gut microbiomes. Nat Microbiol. 2021;6:499–511. 10.1038/s41564-020-00861-0. 33526884 10.1038/s41564-020-00861-0 PMC8007473

[70] Krautkramer K, Fan J, Bäckhed F. Gut microbial metabolites as multi-kingdom intermediates. Nat Rev Microbiol. 2021;19:77–94. 10.1038/s41579-020-0438-4. 32968241 10.1038/s41579-020-0438-4

[71] Greening C, Grinter R. Microbial oxidation of atmospheric trace gases. Nat Rev Microbiol. 2022;20:513–28. 10.1038/s41579-022-00724-x. 35414013 10.1038/s41579-022-00724-x

[72] Greening C, Cabotaje P, Alvarado L, Leung P, Land H, Rodrigues-Oliveira T, et al. Minimal and hybrid hydrogenases are active from archaea. Cell. 2024;187:3357-72.e19. 10.1016/j.cell.2024.05.032. 38866018 10.1016/j.cell.2024.05.032 PMC11216029

[73] Greening C, Geier R, Wang C, Woods L, Morales S, McDonald M, et al. Diverse hydrogen production and consumption pathways influence methane production in ruminants. ISME J. 2019;13:2617–32. 10.1038/s41396-019-0464-2. 31243332 10.1038/s41396-019-0464-2 PMC6776011

[74] Zhou Z, Tran P, Cowley E, Trembath-Reichert E, Anantharaman K. Diversity and ecology of microbial sulfur metabolism. Nat Rev Microbiol. 2025;23:122–40. 10.1038/s41579-024-01104-3. 39420098 10.1038/s41579-024-01104-3

[75] Yang W, Sha Y, Chen X, Liu X, Wang F, Wang J, et al. Effects of the interaction between rumen microbiota density-VFAs-hepatic gluconeogenesis on the adaptability of Tibetan sheep to plateau. Int J Mol Sci. 2024;25:6726. 10.3390/ijms25126726. 38928432 10.3390/ijms25126726 PMC11203870

[76] Xue M, Sun H, Wu X, Liu J, Guan L. Multi-omics reveals that the rumen microbiome and its metabolome together with the host metabolome contribute to individualized dairy cow performance. Microbiome. 2020;8:64. 10.1186/s40168-020-00819-8. 32398126 10.1186/s40168-020-00819-8 PMC7218573

[77] Li Z, Zhao X, Jian L, Wang B, Luo H. Rumen microbial-driven metabolite from grazing lambs potentially regulates body fatty acid metabolism by lipid-related genes in liver. J Anim Sci Biotechnol. 2023;14:39. 10.1186/s40104-022-00823-y. 36879349 10.1186/s40104-022-00823-y PMC9990365

[78] Frampton J, Murphy K, Frost G, Chambers E. Short-chain fatty acids as potential regulators of skeletal muscle metabolism and function. Nat Metab. 2020;2:840–8. 10.1038/s42255-020-0188-7. 32694821 10.1038/s42255-020-0188-7

[79] Glancy B, Balaban R. Energy metabolism design of the striated muscle cell. Physiol Rev. 2021;101:1561–607. 10.1152/physrev.00040.2020. 33733879 10.1152/physrev.00040.2020 PMC8576364

[80] Liu L, Sun D, Mao S, Zhu W, Liu J. Infusion of sodium butyrate promotes rumen papillae growth and enhances expression of genes related to rumen epithelial VFA uptake and metabolism in neonatal twin lambs. J Anim Sci. 2019;97:909–21. 10.1093/jas/sky459. 30535158 10.1093/jas/sky459 PMC6377441

[81] Zhang K, Zhang Y, Qin J, Zhu H, Liu N, Sun D, et al. Early concentrate starter introduction induces rumen epithelial parakeratosis by blocking keratinocyte differentiation with excessive ruminal butyrate accumulation. J Adv Res. 2024;66:71–86. 10.1016/j.jare.2023.12.016. 38128723 10.1016/j.jare.2023.12.016 PMC11674766

[82] Chen C, Yin Y, Li H, Zhou B, Zhou J, Zhou X, et al. Ruminant-specific genes identified using high-quality genome data and their roles in rumen evolution. Sci Bull. 2022;67:825–35. 10.1016/j.scib.2022.01.023. 10.1016/j.scib.2022.01.023 36546235

[83] Na S, Guan L. Understanding the role of rumen epithelial host-microbe interactions in cattle feed efficiency. Anim Nutr. 2022;10:41–53. 10.1016/j.aninu.2022.04.002. 35647325 10.1016/j.aninu.2022.04.002 PMC9117530

[84] Xie Y, Sun H, Xue M, Liu J. Metagenomics reveals differences in microbial composition and metabolic functions in the rumen of dairy cows with different residual feed intake. Anim Microbiome. 2022;4:7. 10.1186/s42523-022-00170-3. 35260198 10.1186/s42523-022-00170-3 PMC8902708

[85] Lin L, Xie F, Sun D, Liu J, Zhu W, Mao S. Ruminal microbiome-host crosstalk stimulates the development of the ruminal epithelium in a lamb model. Microbiome. 2019;7:83. 10.1186/s40168-019-0701-y. 31159860 10.1186/s40168-019-0701-y PMC6547527

[86] Yan X, Si H, Zhu Y, Li S, Han Y, Liu H, et al. Integrated multi-omics of the gastrointestinal microbiome and ruminant host reveals metabolic adaptation underlying early life development. Microbiome. 2022;10:222. 10.1186/s40168-022-01396-8. 36503572 10.1186/s40168-022-01396-8 PMC9743514

