# Supplementary material for: Integrative analysis of rumen microbiota and host multi-organ interactions underlying feed conversion efficiency in Hu sheep
Source: J Anim Sci Biotechnol. 2026 Feb 3;17:19. doi: 10.1186/s40104-025-01333-3 (PMC12865948; doi:10.1186/s40104-025-01333-3)
Supplement: Supplementary file 2 — Additional file 2: Fig. S1. Comprehensive KEGG pathway landscape from differential KOs. Fig. S2. Class-level profiles of CAZymes in HFCR and LFCR groups. Fig. S3. Terminal reductases and hydrogenase subclasses across HFCR and LFCR groups. Fig. S4. Genus-resolved heatmap of terminal reductases and hydrogenase subclasses in HFCR and LFCR rumen metagenomes. Fig. S5. Correlation heatmap between microbial functional genes and host co-expression modules. Fig. S6. Validation of hepatic gene expression by RT-qPCR. [file 40104_2025_1333_MOESM2_ESM.docx]

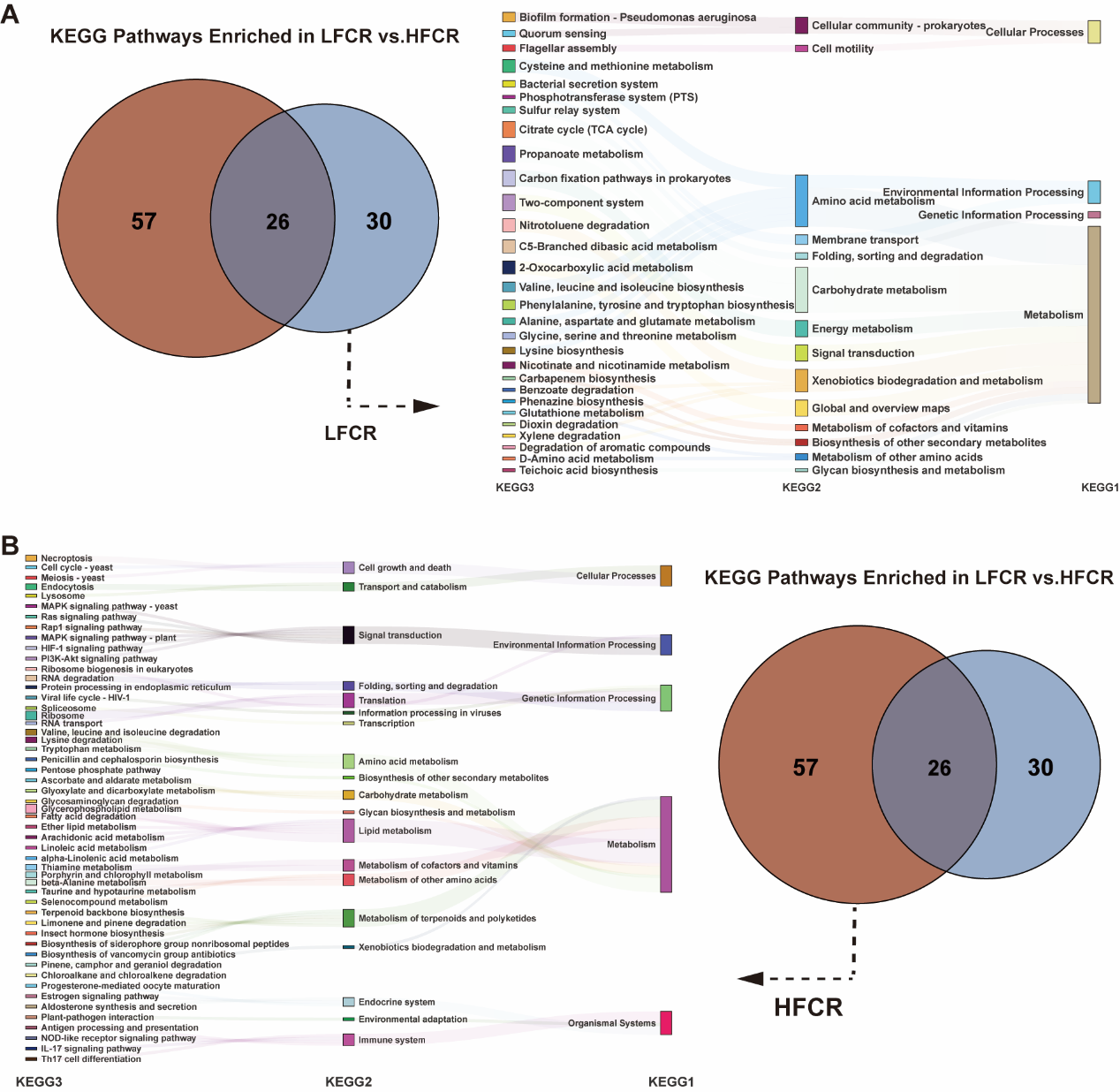


**Figure S1. Comprehensive KEGG pathway landscape from differential KOs. A** LFCR-enriched pathways; **B** HFCR-enriched pathways. Sankey nodes correspond to KEGG level 3 → level 2 → level 1 (left→right). Link widths are proportional to the number of group-enriched KOs assigned to each pathway (*P* < 0.05).

**
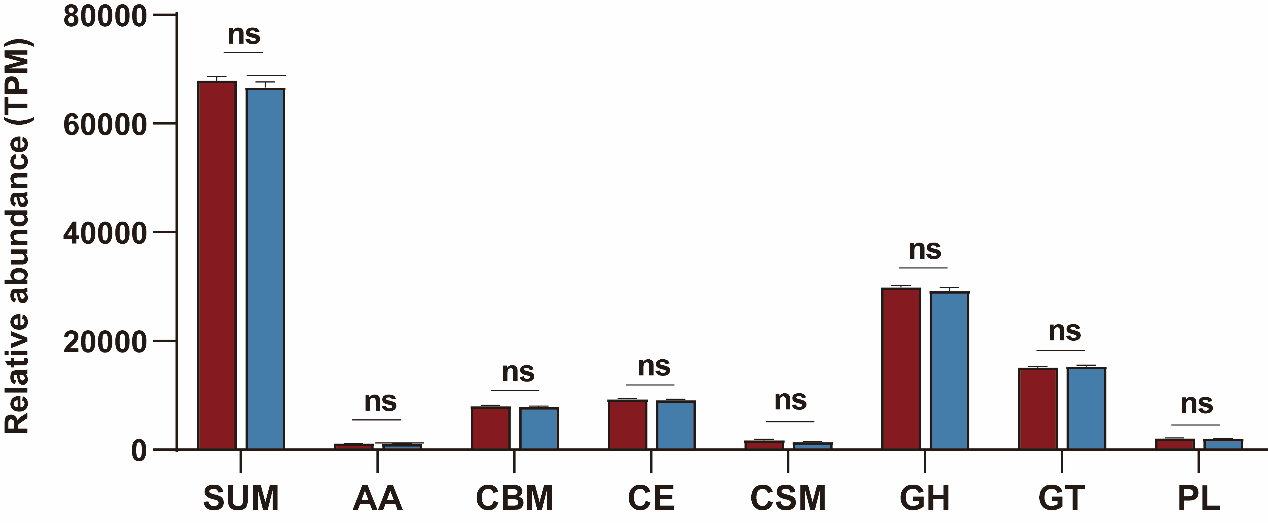
**

**Fig. S2. Class-level profiles of CAZymes in HFCR and LFCR groups.** Relative abundances of major CAZyme classes, including glycoside hydrolases (GH), glycosyltransferases (GT), polysaccharide lyases (PL), carbohydrate esterases (CE), carbohydrate-binding modules (CBM), and auxiliary activities (AA). No significant differences were detected between groups.

**
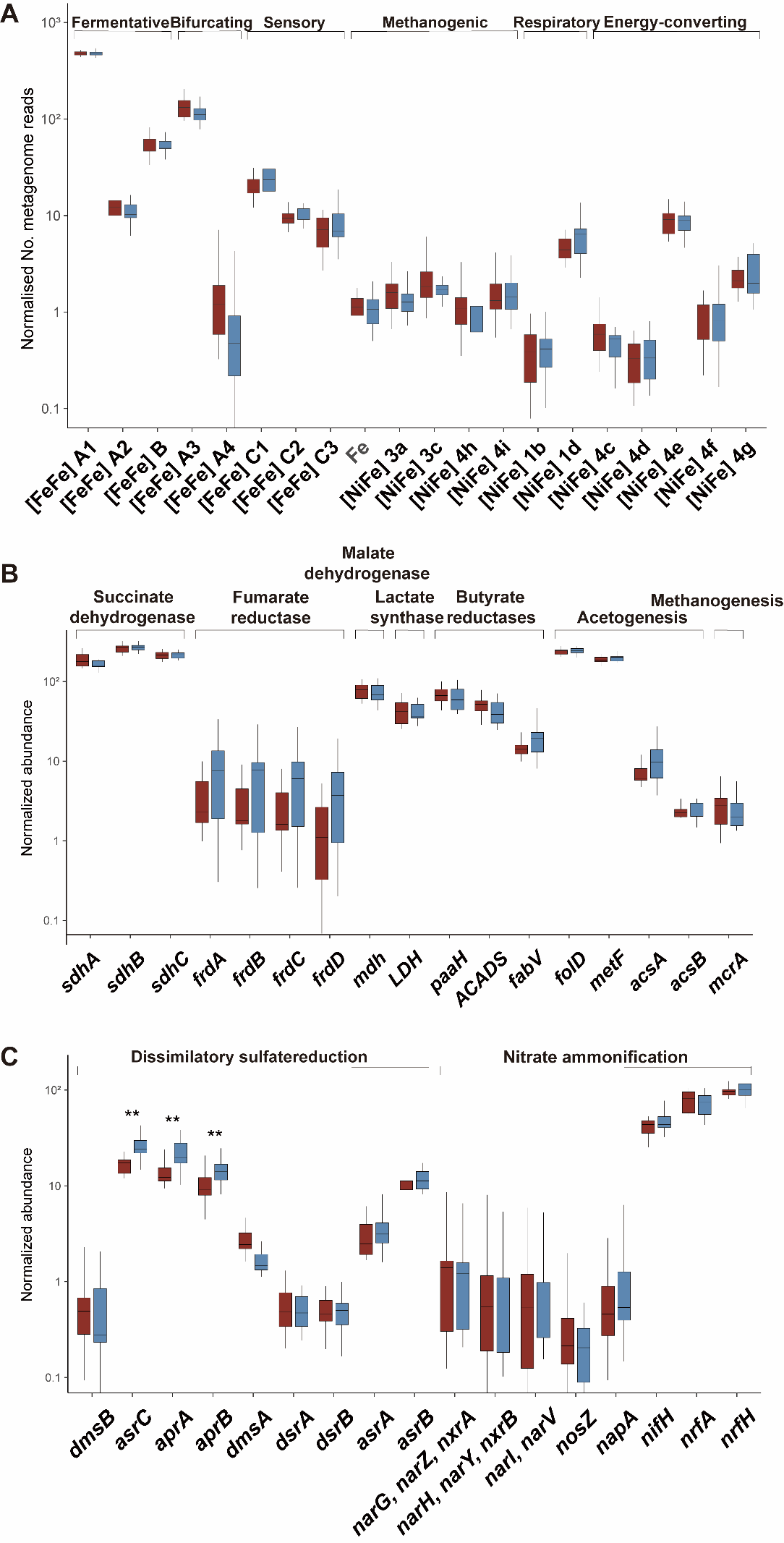
**

**Figure S3. Terminal reductases and hydrogenase subclasses across HFCR and LFCR groups. A** TPM-normalized abundances (log_10_ scale) of genes for fermentative, bifurcating, sensory, methanogenic, respiratory, and energy-converting ([FeFe], [NiFe], [Fe]). **B** Genes from key fermentation and electron-accepting routes: succinate dehydrogenase (*sdhA–C*), fumarate reductase (*frdA–D*), malate dehydrogenase (*mdh*), lactate synthase (LDH), butyrate pathway reductases (e.g., *paaH*, *ACADS*), acetogenesis (*acsB*) and methanogenesis (mcrA). **C** TPM-normalized abundances of genes for dissimilatory sulfate reduction and nitrate ammonification. Boxes show interquartile ranges with medians; whiskers extend to 1.5×IQR. Significance by Wilcoxon rank-sum (nominal): * *P* < 0.05, ** *P* < 0.01. **D** Schematic summarizing inferred electron-flow routes.

**
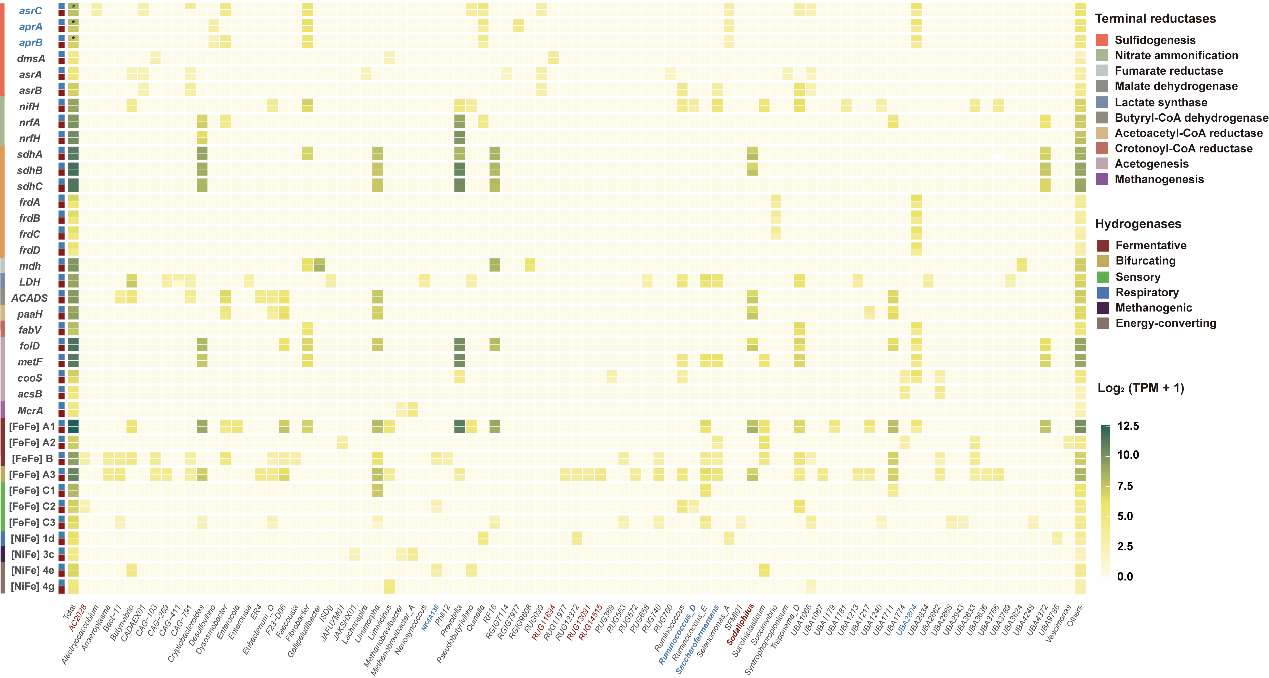
**

**Figure S4. Genus-resolved heatmap of terminal reductases and hydrogenase subclasses in HFCR and LFCR rumen metagenomes.** Rows show terminal-reductase genes and representative hydrogenase subclasses (HydDB); columns represent genera. Cell colors denote genus-assigned abundance (log₂ [TPM + 1]). Left-side bar annotations indicate enzyme category (terminal reductase) and hydrogenase functional class.

**
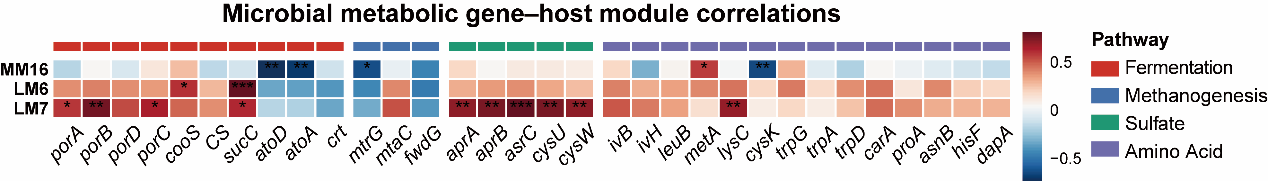
**

**Figure S5. Correlation heatmap between microbial functional genes and host co-expression modules.** Color indicates Spearman correlation coefficient; asterisks denote significance levels (**P* < 0.05, ***P* < 0.01, ***P* < 0.001). Functional categories are color-coded: fermentation (red), hydrogen (blue), sulfate (green), and amino acid (purple).


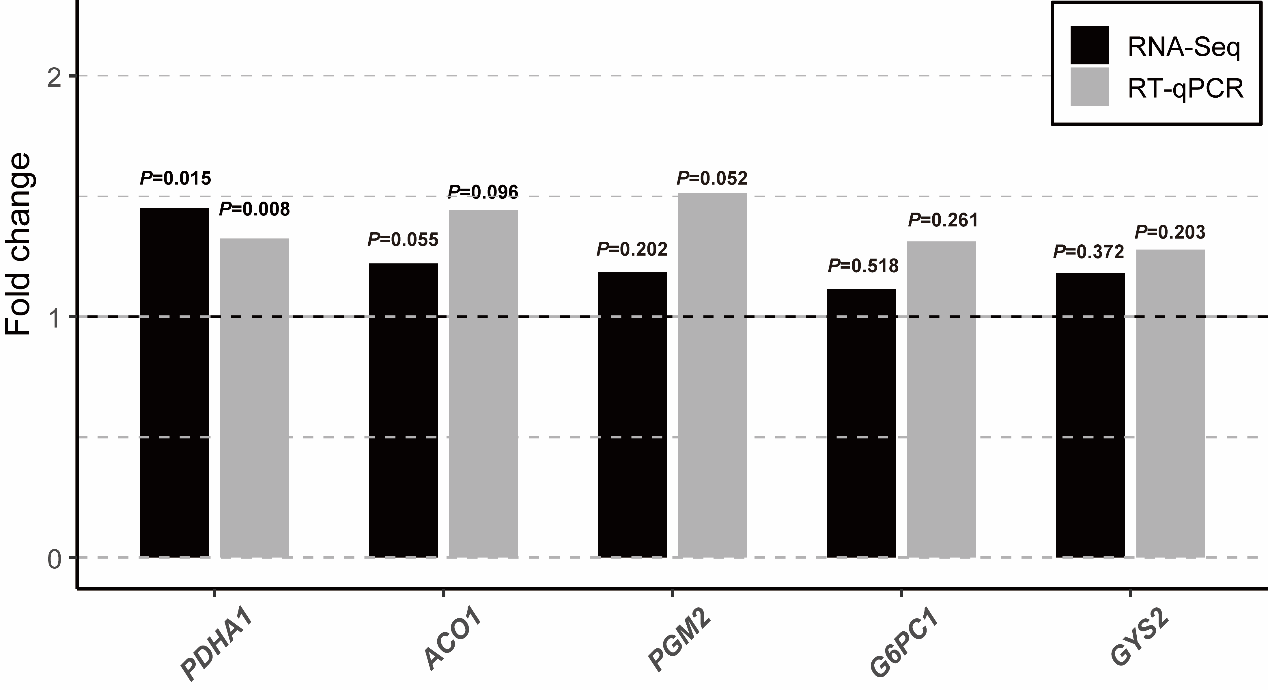


**Fig. S6. Validation of hepatic gene expression by RT-qPCR.** RT-qPCR validation of five hepatic genes (*PDHA1*, *ACO1*, *PGM2*, *G6PC1*, *GYS2*) involved in carbohydrate and energy metabolism. Gene expression changes quantified by RNA-seq (black bars) and RT-qPCR (grey bars) are shown side-by-side. Values represent the fold change of LFCR relative to HFCR. *P*-values above the grey bars indicate statistical differences based on RT-qPCR analysis.
